# TET CpG sequence-context-specific DNA demethylation shapes progression of IDH-mutant gliomas

**DOI:** 10.1016/j.xcrm.2026.102682

**Published:** 2026-03-17

**Authors:** Youri Hoogstrate, Santoesha A. Ghisai, Levi van Hijfte, Rania Head, Iris de Heer, Marta Padovan, Maurice de Wit, Wies R. Vallentgoed, Angelo Dipasquale, Maarten M.J. Wijnenga, Bas Weenink, Rosa Luning, Sybren L.N. Maas, Adela Brzobohata, Michael Weller, Tobias Weiss, Maximilian J. Mair, Anna S. Berghoff, Adelheid Wöhrer, Albert Jeltsch, Johan A.F. Koekkoek, Hans M. Hazelbag, Mathilde C.M. Kouwenhoven, Yongsoo Kim, Bart A. Westerman, Bauke Ylstra, Anneke M. Niers, Kevin C. Johnson, Frederick S. Varn, Roel G.W. Verhaak, Mustafa Khasraw, Martin J. van den Bent, Pieter Wesseling, Pim J. French

**Affiliations:** 1 Department of Neurology, Erasmus MC Cancer Institute, Erasmus MC, Rotterdam, the Netherlands; 2 Department of Neurosurgery, University Clinic Erlangen, Erlangen, Germany; 3 Department of Oncology, Oncology 1, Veneto Institute of Oncology IOV-IRCCS, 35128 Padua, Italy; 4 RCCS Humanitas Research Hospital, Via Alessandro Manzoni 56, Rozzano, Milan, Italy; 5 Department of Pathology, Erasmus MC Cancer Institute Erasmus MC, Rotterdam, the Netherlands; 6 Department of Pathology, Leiden University Medical Center, Leiden, the Netherlands; 7 Department of Neurology, Clinical Neuroscience Center, University Hospital Zurich and University of Zurich, Zurich, Switzerland; 8 Division of Oncology, Department of Medicine I, Medical University of Vienna, Vienna, Austria; 9 Department of Pathology, Neuropathology and Molecular Pathology, Medical University of Innsbruck, Innsbruck, Tyrol, Austria; 10 Division of Neuropathology and Neurochemistry, Department of Neurology, Medical University of Vienna, Vienna, Austria; 11 Institute of Biochemistry and Technical Biochemistry, Department of Biochemistry, University of Stuttgart, Stuttgart, Germany; 12 Department of Neurology, Leiden University Medical Center, Leiden, the Netherlands; 13 Department of Pathology, Haaglanden MC, The Hague, the Netherlands; 14 Department of Neurology, Amsterdam UMC, Amsterdam, the Netherlands; 15 Department of Pathology, Amsterdam UMC, Cancer Center Amsterdam, Amsterdam, the Netherlands; 16 Department of Human Genetics, Amsterdam UMC, Amsterdam, the Netherlands; 17 Department of Neurosurgery, Yale University, New Haven, CT, USA; 18 The Jackson Laboratory for Genomic Medicine, Farmington, CT, USA; 19 Department of Genetics and Genome Sciences, University of Connecticut Health Center, Farmington, CT, USA; 20 Institute for Systems Genomics, University of Connecticut, Storrs, CT, USA; 21 Department of Neurosurgery, Duke University, Durham, NC, USA; 22 Princess Máxima Center for Pediatric Oncology, Utrecht, the Netherlands

**Keywords:** IDH-mutant glioma, oligodendroglioma, tumor evolution, TET, DNA-methylation, sequence context, continuous grading coefficient

## Abstract

Treatment decisions in IDH-mutant oligodendrogliomas are shaped by tumor aggressiveness, underscoring the need for objective grading of these malignant brain tumors. We collect 302 primary and recurrent resections from oligodendrogliomas and perform Ki-67 staining, proteomics, and DNA methylation profiling. During tumor progression, DNA methylation of oligodendrogliomas changes along a continuum. This continuum is linked to increased epigenetic aging, methylation of transcription factors and Ki-67+ cell density, and large-scale DNA demethylation. Demethylation correlates with CpG flanking sequences preferred by TET enzymes. We confirm these findings in previously profiled astrocytomas, indicating IDH-mutant gliomas progress along a shared epigenetic axis. We develop an objective DNA methylation-based prognostic continuous grading coefficient (CGC^ψ^) that captures these changes and outperforms the World Health Organization (WHO) grading for oligodendrogliomas. Our findings underscore the potential of DNA methylation-based grading to more accurately reflect tumor biology and inform clinical decision-making in IDH-mutant gliomas.

## Highlights

• Oligodendrogliomas and astrocytomas progress along a shared epigenetic axis
• A continuous grading coefficient is available as freely accessible computational model
• Sequence context-specific demethylation correlates with TET enzyme sequence preference

## Graphical abstract

**Figure:**
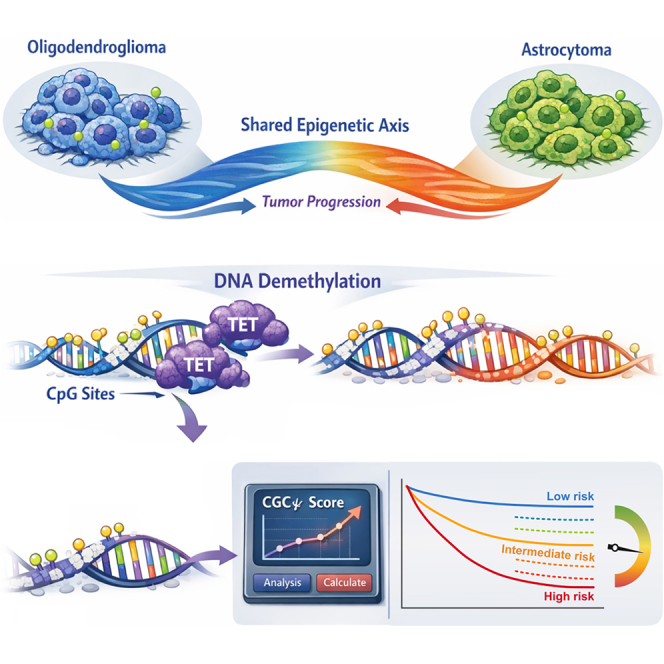

## Teaser

Hoogstrate et al. reveal that IDH-mutant oligodendrogliomas and astrocytomas undergo continuous DNA methylation changes over a shared epigenetic axis during progression. The extent of demethylation across sequence contexts reflects the preferences of TET enzymes demethylating DNA. The prognostic axis of DNA methylation changes is incorporated into a computer application.

## Introduction

Oligodendrogliomas, IDH-mutant and 1p/19q codeleted (“oligodendrogliomas”) are IDH-mutant gliomas defined by a 1p/19q codeletion, typically accompanied by TERT-promoter mutations and retained ATRX expression, distinguishing them from astrocytomas, IDH-mutant (“astrocytomas”).^1^^,^^2^ With a median overall survival of 15 years, the group of patients diagnosed with oligodendroglioma has a more favorable outcome compared to those with other prevalent diffuse glioma types.^3^^,^^4^ Whereas tumor aggressiveness of oligodendroglioma is believed to be a continuum,^5^ in clinical practice, a distinction is made between central nervous system World Health Organization (CNS WHO) grade 2 (grade 2) and CNS WHO grade 3 (grade 3). According to the WHO classification,^1^ this distinction is determined based on histological criteria such as the presence/absence of high mitotic activity, microvascular proliferation, and necrosis. While this classification is used for treatment decision-making, the criteria for grading are not unequivocally defined,^6^ for instance, by the lack of a standardized cutoff for cell division markers Ki-67/MiB or mitotic count.^5^^,^^6^^,^^7^^,^^8^ As a result, there is a high interobserver variability in oligodendroglioma grading.^9^ The difficulty in grading oligodendrogliomas is demonstrated by several recent studies in which no significant difference in prognosis between WHO grades was found,^10^^,^^11^^,^^12^ indicating a need for better and objective ways to define prognosis.

A limited number of imaging and (epi)genetic markers have been linked to the malignant progression of oligodendroglioma, but often not independently validated.^2^^,^^13^^,^^14^^,^^15^^,^^16^^,^^17^^,^^18^^,^^19^^,^^20^ These include contrast enhancement on magnetic resonance imaging^21^^,^^22^; the number of mitoses per mm^2^;^23^^,^^24^ HOX locus hypermethylation,^25^ including *HOXD12*^26^ and *HODX13*^27^; and *CDKN2A/B* homozygous deletions.^8^^,^^18^^,^^28^ A subset of oligodendrogliomas, named “oligosarcoma,” develop an aggressive phenotype with sarcomatous features and are characterized by a distinct DNA methylation profile.^29^ Clinicians often face a dilemma: whether to defer radiotherapy and chemotherapy or to pursue a more aggressive approach. These treatment decisions are based on the anticipated prognosis of patients, and, therefore, more objective robust stratification approaches are required to determine the aggressiveness of oligodendroglial tumors.^30^^,^^31^^,^^32^ Due to its widespread use in neuro-oncology, DNA methylation-based profiling has been proposed to enhance oligodendroglioma grading^11^^,^^33^ and has been applied to assign prognostic features in gliomas.^25^^,^^29^^,^^33^^,^^34^^,^^35^^,^^36^^,^^37^^,^^38^

Studies in which patients and their tumors are followed longitudinally can yield insight into the value of such markers, and into the molecular mechanisms underlying the malignant transformation of gliomas.^34^^,^^39^^,^^40^^,^^41^ We, therefore, established the GLASS-OD workgroup as part of the International Glioma Longitudinal Analysis (GLASS) consortium.^42^ We collected longitudinal tumor samples from 127 patients diagnosed with oligodendroglioma who had undergone more than one surgical intervention with at least 6 months in between and investigated these molecular tumor profiles over time and grade.

## Results

### Primary-recurrent GLASS-OD oligodendroglioma cohort

**Figure 1:**
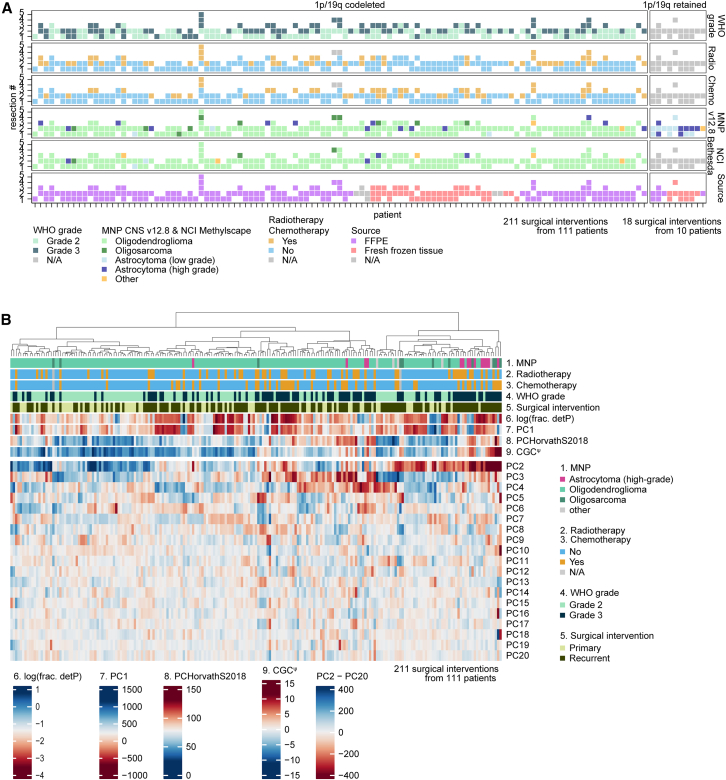
Cohort overview (A) Surgical interventions per patient in the GLASS-OD cohort. Patients are separated in panels on their 1p/19q codeletion status determined by DNA methylation data. The 1p/19q codeletion-negative patients were excluded from further analysis. (B) Heatmap of *n* = 211 1p/19q codeleted surgical interventions combined with patient and sample characteristics clustered on principal components PC2–PC20.

For this study, DNA methylation data were generated from 267 surgical resections obtained from 127 patients. After removal of samples with low tumor purity (<10%) or poor quality, and patients with samples that lacked 1p/19q codeletions, the final discovery dataset consisted of 211 surgical interventions of 111 oligodendroglioma patients from multiple institutions (Figures 1A and 1B; Table S1). Clinical parameters including CNS WHO grade were provided by the respective hospitals. An independent oligodendroglioma validation set, comprising samples from patients who underwent single and multiple surgeries, was assembled from the literature and included 91 tumor samples from 76 patients^11^^,^^29^^,^^43^^,^^44^ (Figures S1A and 1B; Table S2). From the TCGA-LGG dataset,^45^ 150 primary oligodendroglioma samples were obtained, and from the GLASS-NL study,^34^ 203 primary-recurrent astrocytomas were obtained.

### Oligodendrogliomas and astrocytomas progress along a shared epigenetic axis

**Figure 2:**
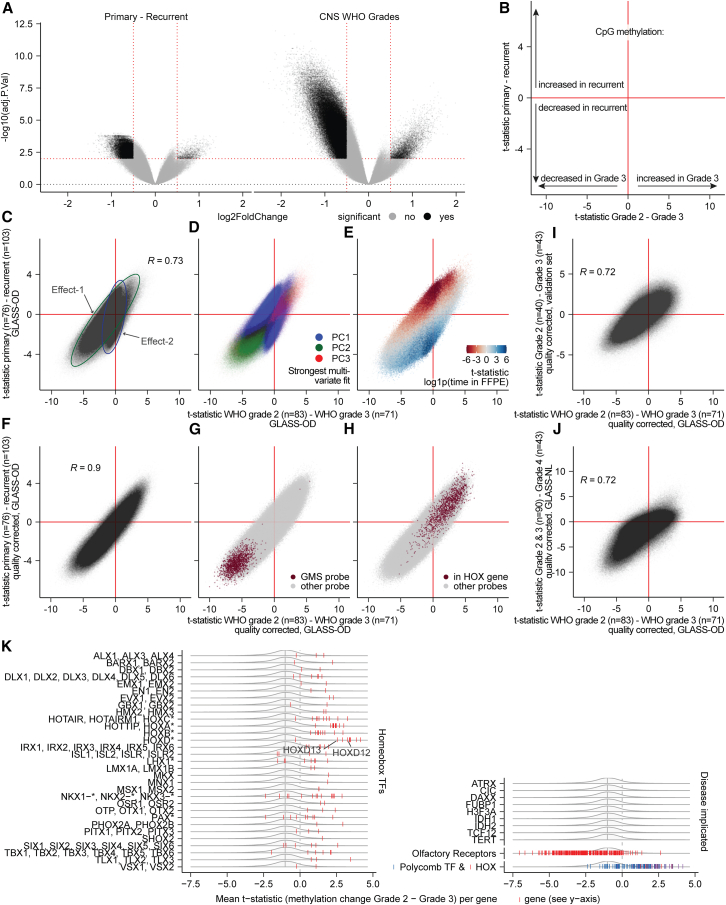
Multiple underlying mechanisms contribute to DNA methylation differences between CNS WHO grades and between primary and recurrent tumors (A) Volcano plots summarizing the DMP analyses, comparing DNA methylation per CpG between primary-recurrent tumor samples (left) and between CNS WHO grades (right). Significant CpGs (q < 0.01, |log2FC|>0.5, empirical Bayes moderated *t* test) are marked in black and non-significant CpGs in gray. Red lines represent the significance thresholds. The *y* axes, −log10(q value), are scaled evenly. (B) Schematic representation of the integration of DMP analyses by their t-statistics. The *x* axis represents the relative methylation change observed between CNS WHO grades. Negative values indicate a decrease, and positive values indicate an increase in methylation in grade 3. The *y* axis represents the relative methylation changes observed between primary versus recurrent tumors, where negative values indicate a decrease and positive values indicate an increase in methylation in recurrent tumors. (C) Integration of the DMP analyses by their t-statistics comparing CNS WHO grades (*x* axis) and primary-recurrent (*y* axis) in the GLASS-OD dataset. Two optical effects are highlighted with ellipses (effect-1: green, effect-2: blue). (D) Same as (C), colored by the principal component each CpG related strongest to in a multivariable model. (E) Same as (C), colored by the t-statistic of an additional DMP model fitting CpG methylation to the duration each tissue sample was stored in FFPE (0 for fresh frozen). (F) Integration of the quality-adjusted DMP analyses by their t-statistics comparing CNS WHO grades (*x* axis) and primary-recurrent (*y* axis) in the GLASS-OD dataset. (G) Same as (F), colored by CpGs within *HOX* genes. (H) Same as (F), colored by CpGs most differentially methylated between primary-recurrent astrocytomas (GLASS-NL). (I) Integration of the quality-corrected DMP analyses comparing between WHO grades in the GLASS-OD dataset (*x* axis) and validation set (*y* axis). Pearson correlation coefficient of the t-statistics is indicated with *R*. (J) Integration of the quality-corrected DMP analyses comparing between WHO grade 2 vs. grade 3 oligodendrogliomas (GLASS-OD) on the *x* axis and WHO grades 2 and 3 vs. grade 4 astrocytomas (GLASS-NL) on the *y* axis. Pearson correlation coefficient of the t-statistics is indicated with *R*. (K) Outcome of quality-corrected DMP analysis comparing between WHO grade in oligodendrogliomas, aggregated at gene level. The *x* axis represents per-gene mean t-statistics comparing methylation levels between WHO grade. The all-gene kernel density with 0.5 quantile is indicated in gray. Per-gene (mean aggregated) t-statistics (*x* axis) are indicated in red, and blue for polycomb TFs.

To characterize oligodendroglioma methylomes along malignant transformation, we performed differential methylated position (DMP) analyses. Aiming to better understand the evolutionary trajectories and the respective implications of CNS WHO grading, we compared both *primary* with *recurrent* tumors and *WHO grade 2* with *grade 3* tumors (Data S1). For patients with more than two surgeries, the primary was compared with the last recurrent sample (Figure S2A). This maximizes the time between surgical resections (median: 67.3 months) and its effects, permitting comparisons of identical or even descending WHO grade. Comparing WHO grade 2 tumors with WHO grade 3 tumors maximizes the effect of malignant phenotypes as defined by neuropathological assessment. Likewise, in case patients had multiple surgical interventions of similar grade, the first grade 2 and/or last grade 3 was chosen (Figure S2A). We compared changes between primary versus recurrent and found a large degree of DNA demethylation at tumor recurrence. A similar, but larger, demethylation was observed when comparing WHO grade 2 versus 3 (Figure 2A).

We intersected the outcomes of both comparisons by their t-statistics, as these are signed like a LogFoldChange and normalized against standard error like a *p* value (Figure 2B). Their outcomes were not only correlated but also indicated two optical underlying differences (“effects”) (*r* = 0.73; Figure 2C). To pinpoint individual factors underlying these effects, we performed principal-component analysis (PCA). We mapped the strongest contribution of each CpG to the first three components, showing that optical effect-2 was represented by CpGs that best fitted the first principal component (PC1) (Figure 2D). For these CpGs, the difference in methylation was more pronounced in the primary versus recurrence comparison than between grades. It was associated with the per-sample fraction detection-*p* value, a metric that represents probe data quality (Figure S2B). Given that effect-2 was more pronounced between primary and recurrent samples, which encompassed the longest time intervals between resections, compared to CNS WHO grades, we suspected that it represented a cytosine deamination artifact resulting from prolonged formalin-fixed paraffin-embedded (FFPE) storage. To address this, we estimated each CpG’s association with the respective time the tissue was stored in FFPE (Figure 2E). This displayed a similar overlap between optical effect-2, PC1, and the association with detection-*p*. Effect-2 was characterized by an increase in methylation at recurrence of probes specifically matching the TA[CpG] sequence and loss of methylation at recurrence of probes with high CpG count, typically of probe type I (Figures S2C–S2E). These findings suggest that (methyl-)cytosine deamination is specific to the CpG’s surrounding sequence with apparent differences between deamination of CpGs and mCpGs. Effect-2 is primarily driven by DNA quality and, therefore, an undesired technical artifact rather than biologically relevant. We repeated the DMP analyses by incorporating quality-associated PC1 to correct for this artifact. Incorporation of this factor almost entirely eliminated *effect-2* (Figure 2F), and t-statistics between the intersected grade- and primary-recurrence comparisons were highly correlated (*r* = 0.90). The CpG sites showing differential methylation were skewed toward genome-wide DNA methylation loss at tumor recurrence and grade 3 (*p* < 0.01, single-sided *t* test comparing t-statistics with μ = 0). This included the earlier reported CpGs with the largest change in DNA methylation between primary and recurrent astrocytomas (Figure 2G).^34^ Of the significant probes, 9.1% showed increased methylation at high grade (q < 0.01 and |log2FC|>1, empirical Bayes moderated *t* test), in which CpGs annotated within *HOX* genes were overrepresented (Figure 2H). The overall differences were confirmed in an independent validation set (*r* = 0.72, *p* < 0.01 chi-square test on significant probes, Figure 2I). Large-scale DNA demethylation combined with increased methylation of *HOX*-gene CpGs has also been observed in IDH-mutant astrocytomas, where they also are associated with tumor grade and recurrence.^25^^,^^34^^,^^46^^,^^47^^,^^48^ We, therefore, aimed to further investigate to what extent the malignant change in DNA methylation of oligodendrogliomas and astrocytomas shows similarities. The comparison between grades in astrocytoma (CNS WHO grade 2 and 3 vs. grade 4, GLASS-NL dataset,^34^ both quality corrected) compared with oligodendrogliomas showed, indeed, that the changes in both tumor subtypes were correlated (*r* = 0.72, *p* < 0.01 chi-square test on significant probes, Figure 2J). These data indicate that both IDH-mutant glial tumor subtypes progress along a shared epigenetic axis.

DNA methylation changes in oligodendrogliomas between WHO grades were investigated at gene level. Virtually all the homeobox transcription factors (TFs) had increased mean methylation, as did polycomb-associated TFs, including members of the reported *HOX* gene loci (Figure 2K).^26^^,^^44^^,^^49^ Conversely, genes of the olfactory receptor family were characterized by methylation loss. From genes implicated in oligodendroglioma, decreased methylation of *TERT* (q = 1.77e^−5^, *t* test) and *ATRX* complex member *DAXX* (q = 2.32e^−19^, *t* test) was observed as grade increased.

### WHO grade-specific methylation changes are TET sequence context specific

**Figure 3:**
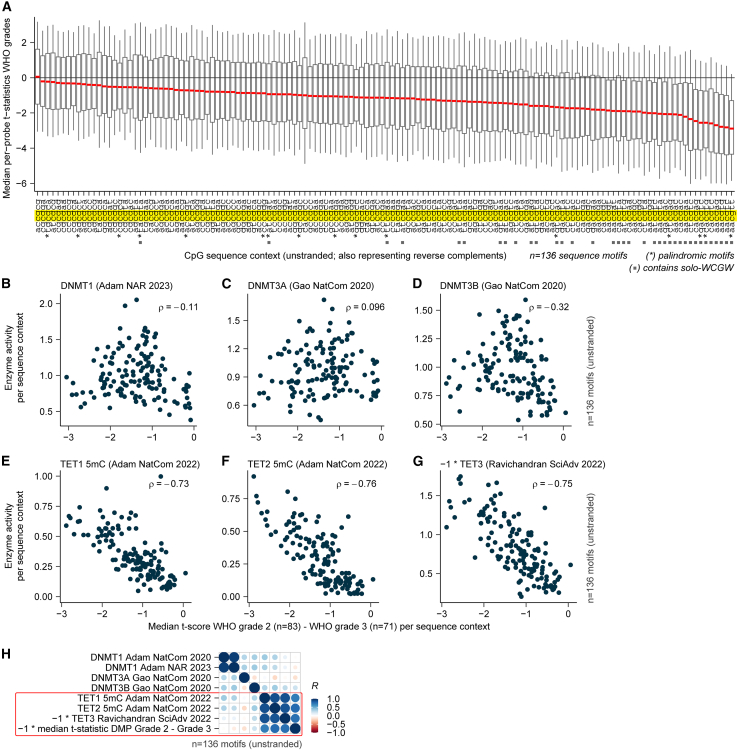
Demethylation is strongest in CpGs with flanking sequences preferred by TET (A). Median t-statistics (between WHO grades, GLASS-OD, quality-corrected; *y* axis) of all CpGs grouped per sequence context (*x* axis). Contexts with identical reverse complement are indicated with an asterisk (∗). Boxplots display the median, the 25th and 75th percentiles, and the 95% confidence intervals. (B–G). Spearman correlation coefficients (ρ) and scatterplots for the median t-statistics per sequence context compared with the (de)methylation preferences of TET and DNMT enzymes. (H). Pearson correlation plot with per-sequence context metrics combined.

Correlated outcomes within oligodendroglioma and between oligodendroglioma and astrocytoma indicate per-CpG specificity and suggest these changes in methylation are not a random process. To explore this further, we mapped the per-CpG change in methylation into bins of CpGs with identical surrounding flanking sequences (the sequence context). Interestingly, we find that the grade-associated DNA demethylation was sequence context specific (Figure 3A). CpGs were more prone to DNA demethylation when flanked by 5′ AA or 3′ TT sequences and more stable in the context of multiple CG di-nucleotides. The sequence contexts that demethylated most are enriched with the “solo-WCGW”-type CpGs, a sequence context linked to oncogenic demethylation and mitotic cell division.^50^ Enzymes from the DNA methyltransferase (DNMT) and ten-eleven translocation methylcytosine dioxygenase (TET) families maintain DNA methylation and exhibit preferences for flanking sequences.^51^^,^^52^ We hypothesized that if such a mechanism is altered, this would affect DNA methylation in a sequence context-specific manner. To address this, we correlated the per-sequence context methylation change between CNS WHO grades with the flanking sequence-dependent activities of DNA (de)methylating TET1-3 and DNMT1-3 enzymes (Figures 3B–3H).^51^^,^^52^^,^^53^^,^^54^^,^^55^^,^^56^^,^^57^^,^^58^ This revealed a strong correlation between demethylation patterns and TET DNA-demethylation flanking sequence preferences (*r* = [–0.73,–0.76]). After applying a correction to the quality effect specific to TA[CpG]NN contexts (Figure S3A), the sequence context-specific demethylation in the astrocytoma dataset displayed a similar correlation with TET enzyme flanking sequence preferences (*r* = [–0.71,–0.86], Figure S3B). These findings demonstrate that grade-associated DNA demethylation in both oligodendrogliomas and astrocytomas is specifically stronger at sites preferential to TET DNA-demethylating enzyme activity.

### CGC^ψ^: Molecular continuous grading of IDH-mutant oligodendrogliomas

To capture malignancy of IDH-mutant astrocytomas reflecting its continuous nature,^34^^,^^59^ we previously developed a DNA methylation based continuous grading coefficient (CGC).^25^ As the underlying probabilities used by CGC are entangled with tumor subtype classification, CGC is astrocytoma specific and does not generalize to other tumor subtypes. Because oligodendrogliomas showed shared temporal changes in methylation compared to astrocytomas, we wanted to assess the presence and prognostic value of CGC in oligodendrogliomas. To this end, we aimed to define a predictor of this grading continuum that is independent of tumor subtype classification. We trained a Least Absolute Shrinkage and Selection Operator (LASSO) regression model on the GLASS-NL IDH-mutant astrocytoma samples, using methylation M-values directly to predict the calculated CGC coefficient for these samples. We used 10-fold cross-validation to assess the performance of predicting CGC, achieving a relative root-mean-square error of 0.352 and Pearson correlation of *r* = 0.94 (Figure S4A). The final model trained on all GLASS-NL astrocytoma samples (CGC^ψ^) consisted of *n* = 168 predicting CpG probes. Among the genes annotated to these CpGs were *WNT1*, *MAPK3*, *HOXA3*, *HOXA6*, *HOXA7*, *HOXA9*, and *HOXC12* (Table S3).

**Figure 4:**
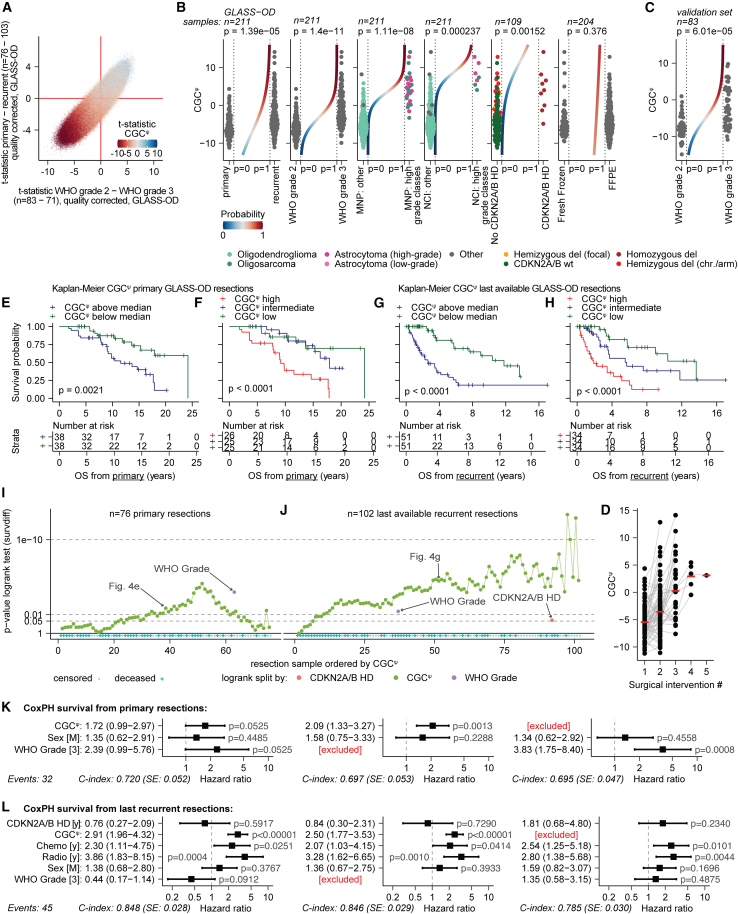
CGC^ψ^ is an objective prognostic continuous grading coefficient for oligodendrogliomas (A) Integrated DMP plot similar as Figure 2F, colored by t-statistics of a DMP model fitting M-values to CGC^ψ^. (B) CGC^ψ^ across various conditions, with a logistic curve in the center. *p* values represent the logistic fit. Conditions (left to right): primary-recurrent, CNS WHO grades, MNP CNS classifier: astrocytoma (high grade) and oligosarcoma vs. other, NCI Methylscape classifier: astrocytoma (high grade) and oligosarcoma vs. other, CDKN2A/B at last available resection, fresh frozen DNA or FFPE. (C) Same as (B) for WHO grade in the validation set. (D) Temporal CGC^ψ^ (*y* axis) per subsequent surgical intervention (*x* axis), with means in red. (E–H) Kaplan-Meier estimates and log rank test *p* values of the overall survival (OS) in the GLASS-OD dataset, splitting either the primary or last available recurrent tumor samples by the mean CGC^ψ^ in two and three groups. (I and J) *p* values from Kaplan-Meier/log rank OS comparisons across a CGC^ψ^-ranked sweep in GLASS-OD. For each cutoff along the CGC^ψ^, a log rank test was performed. Primary tumors (I) and last available recurrent resections (J) were separated. *p* values for WHO grade and CDKN2A/B status (recurrent only) are indicated. Patient censoring is indicated in blue. (K and L) Forest plots and *p* values of multivariate Cox proportional hazards (CoxPH) models for (K) OS from primary resection and (L) post-recurrence survival from the last recurrent resections (GLASS-OD dataset). Panels on the left include both WHO grade and CGC^ψ^, in the center only CGC^ψ^, and on the right only WHO grade. Hazard ratios are provided with 95% confidence intervals.

We applied CGC^ψ^ to GLASS-OD and found the range of scores in oligodendroglioma to be higher than those in IDH-mutant astrocytoma (*p* = 0.015, Wilcoxon test, Figure S4B). To get an indication of how CGC^ψ^ relates to the differences between primary-recurrent tumors and WHO grade in oligodendroglioma, we first estimated the per-CpG association with CGC^ψ^. We then color coded the integrated DMP outcomes accordingly and observed that the CpGs with the largest differences exhibited the strongest association with CGC^ψ^ (Figure 4A). In GLASS-OD, CGC^ψ^ was significantly higher in recurrent tumors, CNS WHO grade 3, Molecular NeuroPathology (MNP) CNS, and NCI Methylscape high-grade classes, and in the presence of CDKN2A/B homozygous deletions (*p* < 0.01, Wald test, range: 1.52e^−3^–1.4e^−11^), but did not differ between FFPE and fresh-frozen samples (*p* = 0.38, Wald test, Figure 4B). In the validation set, CGC^ψ^ was also significantly higher in CNS WHO grade 3 tumors (*p* = 6.01e^−5^, Wald test, Figure 4C). The mean CGC^ψ^ further increased with successive surgical interventions (Figure 4D). These results confirm shared mechanisms of malignant progression in both IDH-mutant tumor types, following a continuum.

Splitting the GLASS-OD cohort by their median CGC^ψ^ for either the primary or the last available resections resulted in CGC^ψ^-high groups with significantly worse survival (*p* = 0.0021; log rank test; Figures 4E and 4F). When splitting into three equally sized groups by ranked CGC^ψ^ score, it displayed a distinct prognosis at recurrence, while in samples from the primary surgery, CGC^ψ^ high had a significantly worse outcome from intermediate and low (Figures 4G and 4H).

To test the prognostic value for each possible cutoff combination, samples were ranked by CGC^ψ^ and the overall survival difference for each cutoff was tested (Figures 4I and 4J). Cox proportional hazard (CoxPH) regression confirmed that in both primary (hazard ratio [HR] = 2.1, *p* < 0.001, 95% confidence interval [CI]: 1.33–3.27, Wald test; Figure 4K) and recurrent tumors (HR = 2.5, *p* < 0.001, 95% CI: 1.77–3.53, Wald test; Figure 4L) CGC^ψ^ was a significant predictor of poor prognosis. Prognosis of CNS WHO grade in models without CGC^ψ^ was, however, significant for primary (HR = 3.8, *p* < 0.001, 95% CI = 1.75–8.4, Wald test, Figure 4K) but not for recurrent tumor samples (*p* = 0.49, Wald test; Figure 4L). In primary tumors (Figure 4K), the concordance index (C-index) of the model that included CGC^ψ^ but not CNS WHO grade (C-index = 0.70) was only marginally higher than CNS WHO grade but not CGC^ψ^ (C-index = 0.69). In primary tumors, CGC^ψ^ and WHO grade lost significance when included together, which in line with their mild variance inflation factors (1.31 and 1.40, respectively) indicated they reduced each other’s unique contribution to the combined model. In recurrent tumors (Figure 4L), the prognostic value of CGC^ψ^ (HR: 2.5, *p* < 0.001; 95% CI: 1.77–3.5; Wald test) was superior to WHO grade (*p* = 0.49, Wald test) and performed independent of WHO grade (*p* < 0.001, Wald test). Both models that included CGC^ψ^ had a higher C-index (= 0.85) than WHO grade alone (C-index = 0.79), showing CGC^ψ^ had superior performance. CoxPH was performed on the last available tumors in the validation set (Figure S4C). Despite the limited number of samples with survival data, CGC^ψ^ was associated with survival (*p* = 0.017, Wald test, C-index = 0.67), while WHO grade was not (*p* = 0.327, Wald test; C-index = 0.58). To further validate CGC^ψ^ in primary tumors, we created a CGC^ψ^-derived model trained only on the intersection of probes present on both the 450k and 850k arrays. Applying this CGC^ψ/450k^ model to the primary oligodendroglioma samples of the TCGA-LGG dataset (Figure S4D) demonstrated more prognostic value (*p* < 0.001, Wald test, C-index = 0.77) than WHO grade (*p* = 0.002, Wald test, C-index = 0.63).

**Figure 5:**
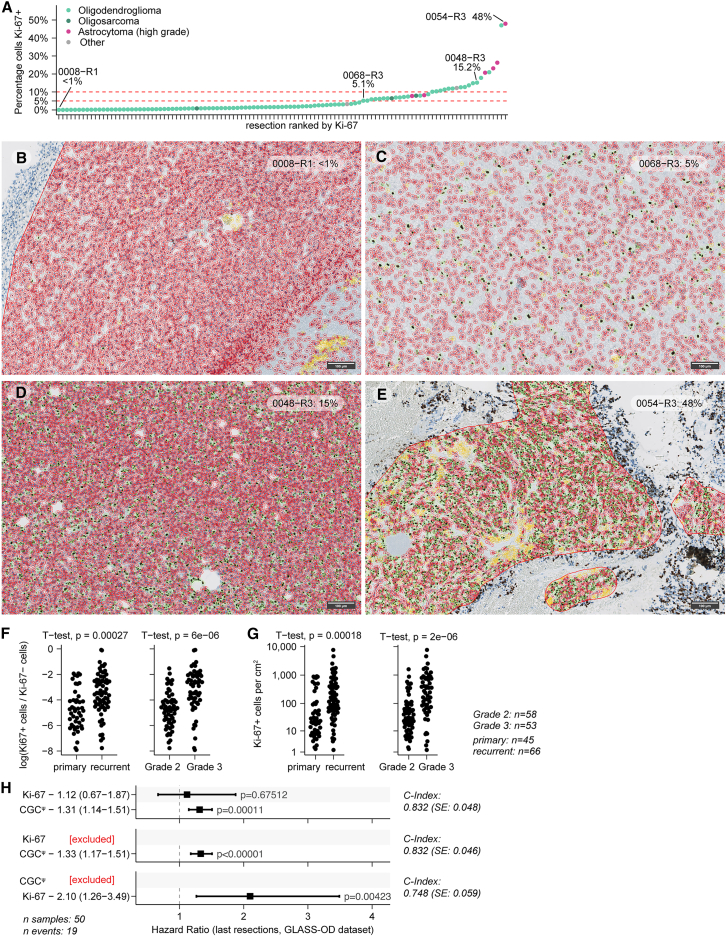
Ki-67-positive cell density is a prognostic factor in oligodendroglioma (A) Percentage of Ki-67 computationally estimated positive cells (*n* = 111 stainings). Tumor samples are on the *x* axis; the percentage on the *y* axis. (B–E) Representative screenshots of computational cell detection and classification of Ki-67 positivity. The percentage of positive cells and sample identifiers are indicated in the top right corner. Detected cells classified as Ki-67 positive are marked in green, Ki-67 negative in red, and artifacts in yellow. Scale bars, 100 μm. (F) Comparison of the log-transformed Ki-67-positive cell ratio across WHO grades (left panel) and between primary and recurrent tumors (right panel). *p* values from two-sided *t* tests are shown on top. (G) Comparison of Ki-67-positive cell density (cells/cm^2^) across WHO grades (left panel) and between primary and recurrent tumors (right panel). *p* values from two-sided *t* tests are shown on top. (H) Overview of multivariate and univariate Cox proportional hazards models with respective *p* values on overall survival (CoxPH) assessing the prognostic value of Ki-67 density at the time of the last available surgical intervention, with Ki-67, CGC^ψ^, or both included. Hazard ratios are indicated with 95% confidence intervals.

Oligodendroglioma samples with sufficient tissue available were stained for Ki-67 (*n* = 111 with matching array). Cells were computationally detected and classified for Ki-67 positivity, resulting in positive cell fractions from <1.0% up to 48.0%, with 67.6% of the samples below 5% positive (Figures 5A–5E; Table S4). Both the Ki-67-positive cell fraction and the density of positive cells per cm^2^ were significantly higher for both samples from CNS WHO grade 3 and recurrent tumors (*p* < 0.0027, Wald test; Figures 5F and 5G). The number of Ki-67-positive cells per cm^2^ was a significant predictor of post-recurrent survival (*p* = 0.004, Wald test, C-index = 0.75; Figure 5H). After incorporation of CGC^ψ^ into the model, Ki-67 lost significance and CGC^ψ^ alone outperformed Ki-67 alone (*p* = 0.004, Wald test; C-index = 0.83; Figure 5H).

### DNA demethylation and accelerated epigenetic aging contribute to the axis of progression

**Figure 6:**
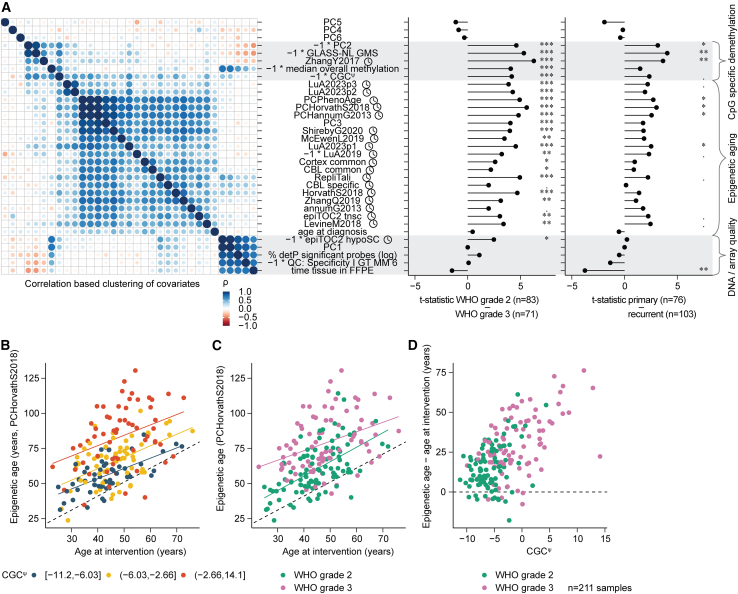
Epigenetic aging is increased as CGC^ψ^ increases (A) Left: Spearman correlation coefficient (ρ)-based clustering of epigenetic clock outputs combined with other sample-level parameters in the GLASS-OD dataset. Right: Linear regression analysis comparing epigenetic clock estimates and additional sample metrics between primary-recurrent and WHO grades. The *x* axis displays t-statistics from linear models comparing metrics across WHO grades (center) and between primary and recurrent tumors (right). False discovery rate-corrected *p* values of empirical Bayes moderated *t* tests are indicated. ⋅q < 0.05; ∗q < 0.01; ∗∗q < 0.001; ∗∗∗q < 0.0001. (B) Concordance between chronological age at surgical intervention (*x* axis) and DNA methylation-based epigenetic age (PCHorvathS2018) (*y* axis). Regression lines indicate three CGC^ψ^ groups defined using the cut function in R. (C) Same as (B), regression lines and colored by WHO grade. (D) Scatterplot of the actual difference between chronological and epigenetic age (PCHorvathS2018, *y* axis) across CGC^ψ^ values (*x* axis).

Of the principal components, PC2 and PC3 differed significantly between CNS WHO grade and primary-recurrent and both correlated with CGC^ψ^ (Figures 6A and 2D). This indicated that beyond quality, two underlying independent factors contribute to the changes in methylation, both of which are captured by CGC^ψ^. PC2 displayed stronger methylation differences between WHO grade and correlated stronger with CGC^ψ^ than PC3. As demethylated CpGs are the predominant contributors to PC2 (Figure 2D), this factor represents predominantly DNA demethylation.

Cellular aging and the cell replicative history within living tissue result in distinct DNA methylation patterns. There are several algorithms able to predict age and cell replicative history (epigenetic clocks) by making use of these patterns.^60^ We applied algorithms provided by metapackage dnaMethyAge^61^ and clustered their outcome with other parameters (Figure 6A, left panel). This was extended with differential comparisons between WHO grade and primary-recurrent (Figure 6A, center and right panels). Of the three resulting clusters, virtually all clocks cluster together. All but two clocks were increased in WHO grade 3 (largest difference in *PCHorvathS2018*, q = 7.06e^−7^, empirical Bayes moderated *t* test). Although it is self-evident that recurrent tumors are epigenetically older than primary, closer inspection of *HorvathS2018* revealed predicted epigenetic ages at recurrence in some cases exceeding 100 years (Figures 6B and 6C), older than their actual age. We observed that epigenetic age indeed exceeded patients’ chronological age at surgical intervention and accelerated specifically as CGC^ψ^ increased (Figure 6D).

PC3 clustered between the epigenetic clocks and displayed strong correlation with *PCHorvathS2018* (ρ = 0.87, Spearman correlation). We further investigated the contribution of all polycomb TF-associated probes to PC3 (Figures 5A and 5B) and found that CpGs within these genes contributed to PC3. For the subset of samples with matching Ki-67 stainings, we estimated correlation with Ki-67 density and positivity fractions with PC2, PC3, and the Horvath epigenetic clock. This revealed that Ki-67-positive cell density was, in particular, correlated with PC3 and the Horvath epigenetic clock (ρ = 0.61–0.68; Figure S5C). Of the two underlying mechanisms, the changes represented by PC3 are related to accelerated epigenetic aging and linked to cell cycling, marked by increased methylation of a broad set of TFs, including those from the *HOX* loci.

### Oligodendroglioma progression is characterized by chromosomal losses

Associations were estimated between per-bin copy number variations (CNV) with primary-recurrent, CNS WHO grades, CGC^ψ^, PC2 and PC3, and post-recurrence survival. Losses at chromosomes 4q, 9p, 13, 14q, 15, and 18 were associated with WHO grade (Figure S6A). When fitting to CGC^ψ^ instead of WHO grade, similar but more pronounced associations were detected (Figure S6B). Interestingly, when fitting to PC2 and PC3, certain genomic alterations fitted uniquely to either PC2 or PC3 (Figures S6C and 6D), suggesting these events contribute to distinct molecular mechanisms. Genomic losses of chr11p showed a trend toward post-recurrence survival (Figure S6E).

We observed a homozygous loss of the *CDKN2A/B* locus in 9.9% (10/111) at the last available tumor sample. Homozygous *CDKN2A/B* deletions were typically focal and spanning the locus specifically. Hemizygous *CDKN2A/B* deletions were more frequent (35/111; 31.5%) and typically encompassed large genomic regions (Figure S7A) as they were mostly partial arm, whole arm, or entire chr9 losses (Table S5). The 9p arm losses were found in 26/111 patients (25.7%) of which four also had a homozygous deletion event. Although the incidence was low (*n* = 10), in multivariable analysis on the last available recurrent tumor (*n* = 101), *CDKN2A/B* HD did not show a statistically significant different overall survival (Figure 4L).

### Oligosarcomas are an aggressive subtype of oligodendroglioma with lower tumor cell fractions

We ran the methylation-based MNP CNS classifier v.12.8 on the GLASS-OD data.^35^ According to this, 14 oligodendroglioma recurrences were high-grade astrocytoma (A_IDH_HG, Figure 1A), of which six had a prediction confidence ≥ 0.84. As indicated in the cohort description, these samples had a 1p/19q codeletion (Figure S8A), and the classifier labeled tumors in the prior resections of these patients as oligodendroglioma. Furthermore, of the 11 samples the classifier v.12.8 labeled as oligosarcoma, 7 were classified as high-grade astrocytoma in v.11.b4, the last version before oligosarcoma was incorporated (Figure S8B). Oligodendrogliomas classified as either oligosarcoma or high-grade astrocytoma were unanimously characterized by the highest CGC^ψ^ scores (*p* = 1.11e^−8^, Wald test, Figure 4B). NCI Methylscape classified four recurrent tumors to be high-grade astrocytoma and three oligosarcoma, which were also characterized by high CGC^ψ^ (*p* = 2.3e^−4^, Wald test, Figure 4B).

**Figure 7:**
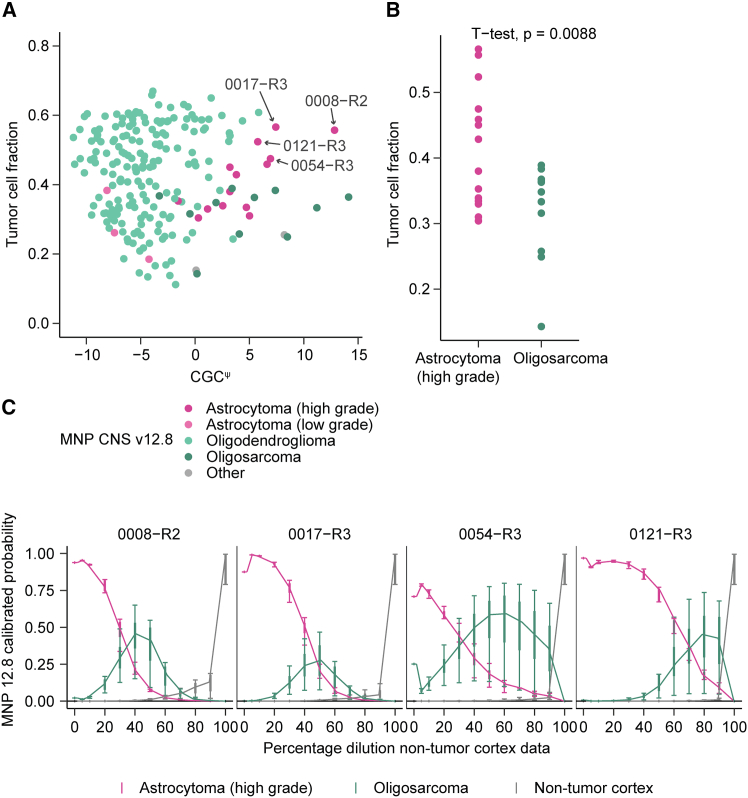
CGC^ψ^ high tumor classification as oligosarcoma or high-grade astrocytoma relates to tumor purity (A) CGC^ψ^ values (*x* axis) versus tumor purity (*y* axis) in GLASS-OD. Samples are color coded by MNP CNS class. (B) Tumor purity of GLASS-OD samples classified as oligosarcoma or high-grade astrocytoma. The *y* axis represents tumor purity, *t* test *p* value on top. (C) *In silico* mixing: four 1p/19q codeleted GLASS-OD samples classified as high-grade astrocytoma were incrementally spiked in with array data from non-tumor cortex and classified using MNP CNS v.12.8. The *x* axis represents the mixed non-tumor fraction. The *y* axis shows the median MNP class prediction probabilities. Vertical bars represent the inner and outer quartiles (thick) and the 95% confidence intervals (whiskers) for the four *in silico* samples.

Our observation that oligodendrogliomas classified as high-grade astrocytoma were characterized by a high CGC^ψ^ could indicate that aggressive astrocytomas and oligodendrogliomas converge toward an indistinguishable overarching epigenetic state. To test this, uniform manifold approximation and projection (UMAP) was performed and showed that oligodendroglioma methylation profiles remained distinguishable from astrocytoma (Figure S8C). Given that oligodendrogliomas remained distinct from astrocytomas, the observation that the methylation-based classification of oligodendrogliomas with high CGC^ψ^ branches into either high-grade astrocytoma or oligosarcoma could indicate diverging evolutionary paths. However, when comparing methylation profiles between the two groups, no CpGs were differentially methylated after multiple-testing correction (Figure S8D). We did find that oligodendrogliomas classified as high-grade astrocytoma were characterized by a significantly higher tumor cell fraction than those classified as oligosarcoma (*p* = 0.009, *t* test, Figures 7A and 7B). To further investigate whether this branched classification fate could be attributed to tumor purity, we performed an *in silico* dilution experiment. Using our software package *idat-tools* (https://github.com/yhoogstrate/idat-tools), we simulated decreasing tumor purity by incrementally spiking in methylation data from non-tumor from whole-brain tissue^62^ samples. In all four tested high-grade astrocytoma-classified oligodendroglioma samples (0017-R3, 0008-R2, 0121-R3, and 0054-R3), incremental fractions of methylation data, indeed, changed the classification fate toward an oligosarcoma diagnosis, until it reached a non-tumor classification (Figures 7C; Table S6). This change in classification was despite the fact that spiked-in non-tumor samples do not fully recapitulate tumor micro-environments. These results indicate that tumor purity played a role in classifying aggressive oligodendrogliomas as high-grade astrocytomas.

### No evidence for treatment-induced DNA methylation changes

As temozolomide (TMZ) and radiotherapy have been implicated in treatment-induced DNA alterations,^63^^,^^64^ we investigated whether these therapies leave detectable signatures in DNA methylation profiles. Due to the limited number of treated WHO grade 2 tumors (4 of 80), differential methylation analyses were restricted to the WHO grade 3 tumors. Comparing tumors treated with TMZ, any chemotherapy (TMZ, Procarbazine, CCNU and Vincristine [PCV], chloroethyl-cyclohexyl-nitrosourea [CCNU], bischloroethyl-nitrosourea [BCNU], cisplatin), or radiotherapy with untreated tumors identified 1, 2, and 0 significantly differentially methylated CpG sites, respectively (Table S7). These findings provide no evidence for apparent therapy-induced methylation patterns.

### Molecular mechanism of oligodendroglioma progression

We performed proteomics on 118 resections with matching methylation data (Data S2). To identify proteins associated with CGC^ψ^, linear regression was performed with CGC^ψ^ as continuous covariate. This resulted in 78/6,563 significant proteins (q < 0.01; |LFC|>0.5, empirical Bayes moderated *t* test, Data S3). Pathway enrichment analysis indicated that proteins involved in (collagen-containing) extracellular matrix (GO:0062023) and adaptive immune response pathways (GO:0002250) had significant increased protein expression (Figure S9A). Expression levels of MAG, MBP, and PLP1, implicated in myelination of oligodendrocytes, decreased when CGC^ψ^ increased, which could point toward dedifferentiation. Integrating the outcome with a similar analysis in the GLASS-NL astrocytomas (Data S4) showed a correlated outcome (*r* = 0.62), including upregulation of collagen-containing extracellular matrix proteins and markers of tumor progression including PCNA and TMPO (Figure S9B).

We conducted differential protein analysis between primary-recurrent and between WHO grade 2 and 3 oligodendrogliomas. Similar to DNA methylation, more differences were identified between grade (*n* = 43) than between primary-recurrent (*n* = 1) (q < 0.01, empirical Bayes moderated *t* test). Of these, *n* = 12 proteins were shared among the 78 differentially expressed proteins across the CGC^ψ^ continuum. Upregulated proteins showed significantly more DNA demethylation at their transcription start site than downregulated proteins (Figure 9C, *p* = 0.0095, *t* test). This inverse relation may be indicative for methylation changes underlying the grade- and recurrent-specific proteome changes.

DNA replication during cell division occurs with locus-specific timing.^50^ Using Repli-Seq, the relative timing of a locus being replicated during cell cycling (early/late replicon) can be measured. We examined whether the extent to which DNA methylation changed between WHO grades in oligodendrogliomas or with CGC^ψ^ was associated with early/late replicons, but did not observe a correlation (*r* = 0.02–0.08; Figure S9D).

## Discussion

Making confident treatment decisions for oligodendroglioma patients means balancing therapy intensity with quality of life, and to do that well, better ways to estimate tumor aggressiveness are needed.^30^ To address this, we assembled a dataset from the GLASS consortium^42^ including both primary and recurrent tumors, to investigate how DNA methylation patterns relate to tumor behavior. Out of this, we developed CGC^ψ^, a continuous scoring tool applicable to oligodendrogliomas that captures tumor malignancy in a more nuanced, objective way than traditional grading. We showed that CGC^ψ^ is robust and reproducible and could help define where along the spectrum of malignancy a tumor falls. Its prognostic value was better or as good as WHO grade. This may be useful in the clinic by helping to determine when to escalate or de-escalate therapy, allowing treatment to be tailored to both prognosis and patient priorities. For example, it could be investigated whether there is a CGC^ψ^ threshold value up to which vorasidenib—assumed to be less effective in more aggressive tumors—provides benefit.

This primary-recurrence study design also showed a notable DNA quality signal, at least partially due to time-dependent storage conditions. The effect was noticeable in samples stored in FFPE for 5 years or longer. This quality effect is probe and sequence specific, typically affecting probes rich in G (guanine) or 5′ TA dinucleotides, which is in line with reduced probe binding due to cytosine deamination. The affected samples were characterized by lower overall intensity and differences in methylated/unmethylated channel intensity, even after normalization.

Beyond the quality effects, methylated levels change similarly—though with varying effect sizes—between primary-recurrent tumors, and between CNS WHO grades in oligodendrogliomas. However, the statistical power of the comparison between grades is considerably stronger than between the primary-recurrent resections, suggesting that this change does not take place in time linearly (as opposed to quality). This aligns with tumor evolution as a sequence of chance events and also implies that prognosis prediction of less-aggressive tumors comes with more uncertainty.

Our results indicated that oligodendroglioma and astrocytoma progress along a shared prognostic epigenetic axis. However, regardless of grade, astrocytomas and oligodendrogliomas remain distinguishable by their methylation profiles and do not evolve into an overarching malignant state. This axis constitutes at least a sequence context-specific decrease in methylation at sites with flanking sequences preferred by TET and increased methylation of polycomb TF genes.^27^ We find the latter to be strongest correlated with epigenetic clocks, further in line with previous reports on the presence of a link between epigenetic aging and CNS WHO grade.^34^ Moreover, we find epigenetic aging at an accelerated pace in the light of CGC^ψ^, increased at recurrence and linked to the Ki-67+ cell fraction and density. This may be explained by CGC^ψ^ capturing cell cycling or replicative history, both of which are increased in more aggressive tumors. CGC^ψ^, however, models a transition from low- to high-grade IDH mutant gliomas constituting at least two underlying factors, of which only one aligns with epigenetic clocks. While methylation changes associated with tumor malignancy were a prominent source of variation in the data, we were not able to pinpoint a radio- or chemotherapy-induced methylation profile, suggesting these treatments do not affect the methylome during course of the disease.

Our results corroborate a poor prognosis of oligosarcomas as they represent, like oligodendrogliomas classified as high-grade astrocytomas, the extremes on the CGC^ψ^. The results further showed that the distinction between high-grade astrocytoma and oligosarcoma is at least partially related to tumor purity, a factor the MNP CNS classifier is known to be sensitive for.^65^ The poor survival for tumors with a high CGC^ψ^, including but not limited to oligosarcomas, justifies prognostication of oligodendrogliomas based on DNA methylation.^25^^,^^65^ Both classifiers misclassified oligodendrogliomas as high-grade astrocytoma, specifically in aggressive recurrent cases with high tumor purity. For potential clinical applications, this should be taken into consideration.

Although we demonstrate that changes in DNA methylation were associated with patient prognosis, it remains to be determined whether this is causal or the effect of tumor progression. The DNA demethylation exhibited CpG specificity between the different oligodendroglioma and astrocytoma datasets. The demethylation is sequence context specific and correlated with TET enzyme flanking sequence preferences. This may be an indication for an active, TET-mediated form of demethylation as the underlying process to IDH-mutant glioma malignant transformation. However, the correlation with Ki-67 and association with solo-WCGW indicated a link with increased proliferation and may reflect a more general mechanism of tumor aggressiveness.^66^ Additional research is needed to determine whether the changes result from passive or active enzymatic DNA demethylation and to what extent this occurs during cell cycling.

The CDKN2A/B homozygous deletion incidence was somewhat higher than the 6.83% in the prospective French network, POLA cohort,^28^ and did not display a significant difference in survival from the last resection, although the mutated sample size was limited. In line with literature,^67^ hemizygous deletions typically have a large genomic span, and, therefore, it is conceivable that the selective advantage of these losses is not driven by *CDKN2A/B* disruption alone.

In summary, we examined DNA methylation profiles of oligodendrogliomas and characterized their malignant phenotypes. The two most prominent changes, global demethylation and increased methylation of specific TFs, are shared with astrocytomas. Subsequently, we developed an astrocytoma-derived continuous grading classifier that is applicable to and prognostic for oligodendrogliomas. This method is not based on nominal prognostic features such as infrequent DNA alterations, but on the basis of a continuous sequence context-specific shift in the DNA methylation profile. The observed link between global demethylation and the preferential flanking sequences of TET demethylating enzymes is important, as it offers a potentially targetable explanation for the initiation and path of demethylation. Further research is needed to demonstrate its clinical relevance because of the retrospective nature of the study.

### Limitations of the study

This study is based on retrospectively collected data, which introduces heterogeneity in patient demographics and treatment regimens. While all patients had undergone two or more surgeries, some samples were excluded due to low purity or quality. For some patients, either a primary or recurrent tumor sample was missing, limiting the ability to correct for patient-specific signals. Patients with multiple resections were preselected to have at least 6 months of overall survival and are generally expected to be fitter, which may introduce bias. Inclusion of both single- and multi-surgery patients could have resulted in a more heterogeneous overall survival distribution, reducing statistical power in survival analyses. The validation set is constrained by a limited number of samples with available survival data, variation in inclusion criteria across datasets, and differences in sample processing batches. Furthermore, it also included patients who underwent only a single surgical resection.

## Resource availability

### Lead contact

Requests for resources/reagents will be fulfilled by the lead contact, Youri Hoogstrate (y.hoogstrate@erasmusmc.nl).

### Materials availability

This study did not generate new unique reagents.

### Data and code availability

• DNA methylation data are available in GEO: GSE297733, proteomics data in PRIDE: PXD070222. Supplementary tables have been deposited at https://zenodo.org/records/17711746.
• Computer code to analyze all data are available at https://github.com/yhoogstrate/glass-od, for estimating CGC^ψ^ at https://github.com/ErasmusMC-Neuro-Oncology/Continuous_Grading_Classifier/, and mixing ∗.idat files at https://github.com/yhoogstrate/idat-tools/.
• Any additional information required to reanalyze the data reported in this work is available from the lead contact upon request.

## Acknowledgments

This work was funded by KWF 2022-4 EXPL/14788; 10.13039/501100002203The Brain Tumour Charity (GN-000765); and 10.13039/501100023452Stichting Hanarth Fonds, The Netherlands. We acknowledge Dr. Abigail Suwala for (meta-)data for the validation set. We acknowledge the Functional Genomics Center Zurich (FGCZ) of University of Zurich and ETH Zurich and Erasmus MC Pathology Research and Trial Service (PARTS) for their services and facilities. We thank Sascha van der Linden for contributing to the graphical abstract.

## Author contributions

Conceptualization: Y.H., S.A.G., L.v.H., M.P., A.D., M.M.J.W., R.L., T.W., M.C.M.K., Y.K., B.Y., P.W., and P.J.F.; formal analysis and investigation: Y.H., S.A.G., L.v.H., R.H., I.d.H., M.P., M.d.W., A.D., A.S.B., and F.S.V.; resources: all (co-)authors; writing (initial): Y.H., S.A.G., P.W., and P.J.F.; review & editing: all (co-)authors; supervision and funding: Y.H., P.W., and P.J.F.

## Declaration of interests

A.S.B. reports research support from Daiichi Sankyo and Roche; honoraria for lectures; consultation/advisory board participation from Roche Bristol-Meyers Squibb, Merck, Daiichi Sankyo, AstraZeneca, CeCaVa, Seagen, Alexion, Servier, Pfizer, and Ygion; and travel support from Roche, Amgen, and AbbVie. M.J.M. reports research funding from Bristol-Myers Squibb and travel support from Pierre Fabre. M.M.J.W. reports consultancy fee from Servier. M.P. reports involvement with Bayer (invited speaker), Health4U (invited speaker), and Novocure (advisory board). M.W. reports research grants from Novartis, Quercis, and Versameb and honoraria for lectures or advisory board participation or consulting from Anheart, Bayer, Curevac, Hemerion, Iqvia, Medac, Novartis, Novocure, Orbus, Pfizer, Philogen, Roche, and Servier. T.W. reports honoraria from Philogen S.p.A. and research grants from Cellis.

## STAR★Methods

### Key resources table

**Table:** 

REAGENT or RESOURCE	SOURCE	IDENTIFIER
**Antibodies**		

MIB1/Ki-67 (Rabbit anti-human)	Ventana	Cat#790-4286; RRID: AB_2631262

**Critical commercial assays**		

Illumina Infinium MethylationEPIC BeadChip v1.0 (850k)	Illumina	Cat#WG-317-1003
QIAamp DNA FFPE Tissue Kit	Qiagen	Cat#56404
ultraView Universal DAB Detection Kit	Roche	Cat#760-500

**Deposited data**		

DNA Methylation data (GLASS-OD)	This paper	GEO: GSE297733
Proteomics raw data (GLASS-OD)	This paper	PRIDE: PXD070222
DNA Methylation differential analysis outcomes	This paper	Data S1: Zenodo: https://zenodo.org/records/1771174
Proteomics processed data (GLASS-OD)	This paper	Data S2: Zenodo: https://zenodo.org/records/17711746
Proteomics differential analysis outcomes (GLASS-OD)	This paper	Data S3: Zenodo: https://zenodo.org/records/17711746
Proteomics differential analysis outcomes (GLASS-NL)	This paper	Data S4: Zenodo: https://zenodo.org/records/17711746
DNA Methylation data (Mair & Berghoff)	https://doi.org/10.1158/1078-0432.CCR-22-1133	GEO: GSE209579
DNA Methylation data (Suwala & Reuss)	https://doi.org/10.1007/s00401-021-02395-z	GEO: GSE190365
DNA Methylation data (Hervás-Corpión & Valor)	https://doi.org/10.3390/cells12030374	GEO: GSE147391
DNA Methylation data (Malta & Noushmehr)	https://doi.org/10.1158/0008–5472.CAN-23-2093	GEO: GSE248471
DNA Methylation data (TCGA-LGG)	https://doi.org/10.1016/j.cell.2015.12.028	TCGA: TCGA-LGG
DNA Methylation metadata (TCGA-LGG)	https://portal.gdc.cancer.gov/projects/TCGA-LGG	TCGA: TCGA-LGG
DNA Methylation metadata (TCGA-LGG)	https://doi.org/10.1016/j.cell.2015.12.028	TCGA: TCGA-LGG
DNA Methylation data (Vallentgoed & French)	https://doi.org/10.1038/s43018-025-01023-z	EGA: EGAS00001007546
Proteomics data (Vallentgoed & French)	https://doi.org/10.1038/s43018-025-01023-z	PRIDE: PXD062328
RepliSeq data track	https://genome.ucsc.edu/	UCSC: wgEncodeUwRepliSeq

**Software and algorithms**		

GLASS-OD analysis code	This paper	https://github.com/yhoogstrate/glass-od
CGC^ψ^	This paper	https://github.com/ErasmusMC-Neuro-Oncology/Continuous_Grading_Classifier/
DABnn6 [Ki-67+/Ki-67-/artifact] imaging classifier	This paper	https://github.com/yhoogstrate/glass-od/tree/main/QuPath/classifiers/object_classifiers
idat-tools	This paper	https://github.com/yhoogstrate/idat-tools/
MNP CNS PredictBrain classifier	https://doi.org/10.1038/nature26000	https://www.molecularneuropathology.org/
MNP CNS PredictBrain classifier (v12.8)	https://doi.org/10.1016/j.ccell.2025.11.002	https://www.molecularneuropathology.org/
CoNuMee/cnvp_v5.2	https://doi.org/10.1093/bioinformatics/btae029	https://www.molecularneuropathology.org/
NCI Methylscape Bethesda classifier	NCI	https://methylscape.ccr.cancer.gov/
QuPath v0.5.0	https://doi.org/10.1038/s41598-017-17204-5	https://qupath.github.io/
StarDist extension v0.5.0	https://doi.org/10.1007/978-3-030-00934-2_30	https://github.com/stardist/stardist
ImageJ v1.54k	https://doi.org/10.1038/nmeth.2089	https://imagej.net/
infinium-methylationepic-v-1-0-b5-manifest-file.csv	Illumina	https://support.illumina.com/downloads/infinium-methylationepic-v1-0-product-files.html
Prolfqua	https://doi.org/10.1021/acs.jproteome.2c00441	https://github.com/fgcz/prolfqua
R v4.4.2	The R Project for Statistical Computing	https://www.r-project.org/
Limma	https://doi.org/10.1093/nar/gkv007	https://bioconductor.org/packages/release/bioc/html/limma.html
Minfi	https://doi.org/10.1093/bioinformatics/btu049	https://bioconductor.posit.co/packages/release/bioc/html/minfi.html
g:Profiler web portal	https://doi.org/10.1093/nar/gkw199	https://biit.cs.ut.ee/gprofiler/gost
dnaMethyAge	https://doi.org/10.1007/s11357-023-00871-w	https://github.com/yiluyucheng/dnaMethyAge
EpiTOC2	https://doi.org/10.1186/s13073-020-00752-3	https://zenodo.org/records/2632938
RepliTali	https://doi.org/10.1038/s41467-022-34268-8	https://zenodo.org/records/7108429
ComplexHeatmap	https://doi.org/10.1093/bioinformatics/btw313	https://bioconductor.org/packages/release/bioc/html/ComplexHeatmap.html

### Experimental model and study participant details

#### GLASS-OD discovery dataset

Patients histologically diagnosed with oligodendroglioma, IDH-mutant and 1p/19q codeleted, who had undergone more than one surgical intervention with at least six months between the procedures, were eligible for inclusion. Patients were included based on histological diagnosis. Tumor material and detailed clinical follow-up data were collected from institutes in Rotterdam, Amsterdam, Leiden, The Hague, and Utrecht (The Netherlands), as well as in Milan and Padova (Italy), and Durham (Duke University Medical Center [DUMC], USA). From DUMC, isolated DNA from fresh-frozen tissue was made available. From UMC Utrecht, processed DNA methylation arrays derived from FFPE material were available. Clinical parameters are provided in Table S1. Written informed consent was obtained from each subject. These studies were approved by the ethical board of the Erasmus MC (MEC-2020-0087, Rotterdam, The Netherlands), and conducted in accordance with institutional and national regulations.

#### Validation dataset

A validation cohort was assembled from primary and/or recurrent oligodendrogliomas included from publicly available studies with distinct study designs. This cohort was complemented with three in-house oligodendroglioma samples resected only once, falling under the ethical approvement described for the discovery dataset. Clinical parameters are provided in Table S2. Samples were only included when they were processed using the Illumina BeadChip 850k V1 platform. As no sufficiently sized primary–recurrent EPIC 850k V1 dataset was available, we included oligodendroglioma samples regardless of the number of surgeries the patients had undergone. Based on CoNuMee CNV profiles, samples with insufficient estimated purity (<10%) or lacking 1p/19q codeletion were excluded. WHO tumor grades were obtained from original pathology reports or corresponding manuscripts and were translated into CNS WHO grades using Arabic numerals instead of Roman numerals.

### Method details

#### Methylation array processing

For the majority of GLASS-OD samples, tissue was received as FFPE blocks or slides. The FFPE blocks were sectioned into 10 μm slices for DNA isolation, and 4 to 5 additional 10 μm sections were used for hematoxylin and eosin (H&E) staining. From these stainings, regions with the highest fraction of neoplastic cells were identified and marked on the sections by central neuropathologist JMK. These regions were then macrodissected and used for DNA isolation using the QIAamp DNA FFPE Tissue Kit (Qiagen, Cat#56404). Both these samples and those received as isolated DNA were processed using Illumina’s Infinium MethylationEPIC v1.0 BeadChip (850k, Illumina, Cat#WG-317-1003) at the internal facility of the Erasmus MC.

#### TCGA-LGG (1p/19q codel)

We obtained all TCGA-LGG ∗.idat files from https://portal.gdc.cancer.gov/projects/TCGA-LGG. Survival data and 1p/19q codeletion status were obtained from the literature.^45^ Only ∗.idat files from primary samples with matching entries labeled as 1p/19q codeleted were included. Additional survival data were obtained using TCGAbiolinks.^68^ Survival data from TCGAbiolinks were converted from months to days by multiplying by 30.43686. Due to incomplete survival data in both sources, survival data (in days) were intersected. In cases where survival data were available from both sources, the TCGAbiolinks data were used, as they were more up to date (Table S8). In total, 150 ∗.idat samples with survival data were available, of which 21 had a recorded survival event.

#### MNP CNS classifier

Data were classified via the MNP portal using the PredictBrain classifier.^35^ The CoNuMee package,^69^ as incorporated in MNP classifier v12.8,^70^ was used to determine copy number variations (CNVs). Quality control (QC) metrics were obtained from classifier version v11b4. For 10 out of 121 patients in the discovery dataset, resections were misclassified as astrocytoma due to the absence of a detectable 1p/19q codeletion. These cases were excluded from all subsequent analyses. The final GLASS-OD methylation cohort consists of 211 resections from 111 patients.

#### GLASS-NL dataset

Astrocytoma samples from the GLASS-NL study were obtained.^34^ Samples with a fraction of det-P failed probes greater than 2.5% were excluded, resulting in 219 samples retained for analysis. These were used for principal component analysis (PCA). Principal component 3 (PC3) correlated with tumor purity and segregated samples with a flat CNV profile. Of the 219 samples, those with a PC3 value below 300 were retained (*n* = 203).

#### DNA methylation processing and analysis

To evaluate sample and probe quality, all ∗.idat files were loaded using minfi 3.18^71^ in R and pre-processed with offset = 0, dyeCorr = T and dyeMethod = "single". Dye correction was set to single to ensure normalization outcome is independent of the poorest quality sample of the run(s) and comparable between runs. While minfi loaded all *n* = 865,859 probes, only the *n* = 760,405 probes not annotated as MASK_general = TRUE were exported to M-values. Detection-P (det-P) fractions were estimated using: detectionP(type = "m+u"). Probes with an insignificant *p*-value (*p*-value >0.01) were marked as failed and the per-probe and per-sample fraction of failed probes was estimated. Low-performing samples and probes were identified by a combination of per-sample det-P failed probes and principal component analysis used only for QC analysis, upon the 200,000 most variable (stats::mad) M-values. By visualizing the per sample percentage det-P value with the correlated principal component, PC1, we exclude samples with a det-P fraction of ≥ 0.025 probes or a PC1 ≥ 875. After tumor purity and quality control, DNA methylation data was available for 229 resections from 121 patients. Copy-number plots were examined for evidence of 1p/19q codeletion. No codeletion was detected in 18 samples from 10 patients, and these cases were excluded from further analysis.

Of these 211 samples and *n* = 685,271 probes passing quality control, resulting M-values (minfi::ratioConvert( …, what = "M")) were exported to a cache file. All post-qc analyses were only conducted on these *n* = 685,271 probes that had not failed detection-P, PCA, targeted specifically CpGs, of the 760.405 probes not annotated as MASK_general = TRUE, except for external tools which required all probes for input (epigenetic clocks and MNP classifier). Beta-values were estimated (minfi::ratioConvert( …, what = "beta")) for external epigenetic clock packages and estimation of per-sample mean/median methylation levels. For sample-to-patient fingerprinting analysis, beta-values of SNP covering probes were extracted (minfi::getSnpBeta(RGSet)) and scaled (scale(, center = F)) before calculating the sample-to-sample Euclidean distance. A post-qc principal component analysis (PCA) was performed on the M-values of GLASS-OD and GLASS-NL datasets, separately. The heatmap including all 1p/19q surgical interventions was clustered on these principal components PC2 - PC20. PC1 was visualized but excluded from this clustering because of its strong associations with quality. UMAP was performed on the top 7,500 most variable CpG probes (mean absolute deviation), not located on chromosomes X, Y and M, using the uwot library. Samples from the validation set were mapped onto the GLASS-OD PCA using the predict() function in R. For gene-level interpretation of DNA methylation in a differential model, the mean t-statistic for all DNA methylation probes per gene were compared with the distribution of the mean t-statistics for the remaining genes. Linear regression analysis was performed using limma to find per-bin copy-number variations associated with malignant progression. In the CNV analyses, tumor purity was incorporated as continuous covariate in the respective regression models as well as per-patient correction. GLASS-OD samples were clustered using ComplexHeatmap.^72^ Tumor purity estimates were plotted along their CoNuMee/CNVP estimated profile.

#### Tumor purity

For oligodendrogliomas tumor purity was estimated using the CNV foldChange (Connumee CNVP v5.2 segment calls) for the 1p/19q codeletion. The respective foldChanges for all genomic bins at chromosomal arms 1p and 19q were used for a 1p/19q codeletion specific principal component analysis, used to select features for purity estimation. The first component was associated with the intensities of the codeletions from both chromosomal arms. From these bins, only the 1,013 bins with PC1 loadings between −0.021 and −0.0345 were selected (Table S9). Then from these bins strongly contributing to PC1, the median foldChange *f* was calculated. Tumor purity *p* was defined as: *p* = −2 ∗ (2^*f*^ – 1). Array samples with a purity below 0.1 (10%), of which none was classified as oligodendroglioma by MNP, were excluded from further analysis. In the validation cohort, 5 samples were excluded due to low purity (Figure S1A). For astrocytomas, PC3 related to tumor purity as it segregated samples with a flat CNV profile. Of the 219 samples, those with a PC3 value smaller than 300 were kept (*n* = 203).

#### Epigenetic clocks

Prior to running epigenetic clock algorithms, samples were first normalized (minfi::preprocessNoob(RGSet, offset = 0, dyeCorr = T, dyeMethod = "single")), exported to beta-values and inserted in epigenetic clock algorithms using the dnaMethyAge metapackage,^61^ including the following clocks: HannumG2013 (human whole blood), LevineM2018 (human whole blood), ZhangQ2019 (human blood and saliva), ShirebyG2020 (human cortex), ZhangY2017 (fitted to time to death; human blood of patients with 38 diseases, including cancer), LuA2019 (human whole blood), HorvathS2018 & PCHorvathS2018 (multiple human cell types; non cancer), McEwenL2019 (buccal epithelial cells), CBL_specific, PCHorvathS2013 (multiple human cell types; non cancer), PCHannumG2013 (human whole blood), PCPhenoAge, CBL_common, Cortex_common, LuA2023p1 & LuA2023p2 & LuA2023p3 (multiple tissue types across multiple mammalian species), YangZ2016 (whole blood). EpiTOC2^60^ and RepliTali^73^ were executed using their original package.

#### CGC^ψ^

CGC^ψ^ was generated as LASSO model (glmnet library) and was trained on the GLASS-NL dataset with CGC, formally CGC=log(p_[A_IDH_LG]_/p_[A_IDH_HG]_),^25^ as response variable, with alpha = 1 and lambda = 0.1041977. The input for CGC^ψ^ prediction was the M-value matrix of the 685,271 probes (offset = 0, dyeCorr = TRUE, dyeMethod = "single") passing quality control, of the 203 GLASS-NL samples passing quality control. For the TCGA-LGG dataset, a derivative model was trained using only the intersection of 685,271 850k probes and 450k array probes. This model was then applied to the TCGA-LGG 1p/19q samples only. A small R package was written to apply the linear predictors: https://github.com/ErasmusMC-Neuro-Oncology/Continuous_Grading_Classifier/tree/v2_with_OD

#### Mixing idat files: idat-tools

For mixing oligodendroglioma ∗.idat files classified as high grade astrocytoma by the MNP CNS classifier with incremental fractions of non-tumor sample, we developed a free open-source software application in python3, idat-tools: https://github.com/yhoogstrate/idat-tools/. It can read an ∗.idat file into memory, change its contents, and write the memory object back to a new ∗.idat file. By mixing the ∗.idat file with a second ∗.idat file, given a fraction (0.0–1.0), all data, including idat columns std_dev, n_probes and intensity, are proportionally mixed, rounded and exported. For ∗.idat files not having an identical number of probes, the intersection of probes is taken. In this study, samples were mixed with fractions {0.1, 0.2, … 0.8, 0.9} (Table S6).

#### Differential methylated position analysis

Differential methylated position analyses were performed by (typically multi-variate) linear modeling of the M-values using limma.^74^ M-values were chosen instead of beta-values due to their symmetrical distribution centered around zero and their unbounded range, both of which make them more suitable for linear modeling. Because earlier DNA methylation profiling of oligodendrogliomas reported the presence of patient-specific effects,^75^ we aimed to factor these out by incorporating patient identifiers in multivariate models. For each experimental design, the included single-patient/patient-unique samples were group into a decoy “remainder” patient. Multivariate models were generated using model.matrix (e.g., model.matrix(∼factor(patient) + factor(primary.recurrence), data = …)), then fitted using lmFit and tested using limma::eBayes(…, trend = T). The appropriate coefficients were exported with limma::topTable (e.g., limma::topTable( …, n = nrow(…), coef = "factor(primary.recurrence)recurrence", sort.by = "none", adjust.method = "fdr")). For visualizations, the subsequent t-statistics were used as they are signed and continuous unlike *p*-values, and corrected for standard error, unlike the log2foldChange. CpG probes were considered significantly different with an FDR adjusted *p*-value <0.01 and a |log2FC| > 0.5. For multivariate models, incorporated continuous factors such as CGC^ψ^ were typically scaled to a standard deviation of 1.

#### Polycomb gene annotations

Genes attributed to Polycomb complexes were taken from the literature.^76^

#### Gene enrichment

The GencodeCompV12_NAME column from Illumina’s infinium-methylationepic-v-1-0-b5-manifest-file.csv manifest was used for probe-to-gene annotation. For probes annotated to more than one gene, the annotation was split and the table was expanded. Genes starting with “RP” followed by numbers were excluded. For each gene, the median t-statistics (between WHO grades) of all probes belonging to the gene was computed and compared with the median t-statistics of other genes. Genes with HUGO symbols starting with ‘OR’ followed by a number (regex: “ˆOR[0–9]”) were considered olfactory receptor family genes.

#### RepliSeq

Processed replication timing experiment results were downloaded from UCSC’s goldenpath wgEncodeUwRepliSeq track(bigWigs files, hg19).^77^ These were converted to hg38 using CrossMap^78^ and exported to bedgraph. CpGs were annotated with their respective replication-seq timing value by intersection with the lifted-over bigWig bins using their genomic coordinates and the HTSeq library in python3.^79^ Excessive outliers in RepliSeq score were excluded (keep: BjWaveSignalRep2 < 79; Bg02esWaveSignalRep1 < 90; BjWaveSignalRep1 < 95; BjWaveSignalRep1 > 10; NhekWaveSignalRep1 < 80). Spearman correlation was estimated between the per-CpG change in methylation between WHO grades with the respective RepliSeq values.

#### Sequence contexts

Probes were mapped to their sequence context deduced from “infinium-methylationepic-v-1-0-b5-manifest-file.csv”. Probe sequences “AlleleA_ProbeSeq” were compared with the genomic alignment sequence “Forward_Sequence” to determine whether the probe’s orientation is forward. The Forward_Sequence was used to extract the sequence context and when probes are in reversed orientation, the reverse complement of the Forward_Sequence was used. The probe to sequence context mappings are available in Data S1. TET and DNMT enzyme flanking sequence preferences were combined from literature.^51^^,^^52^^,^^53^^,^^54^^,^^55^^,^^56^^,^^57^^,^^58^ Both t-statistics and enzyme affinities were integrated from their 256 stranded sequence context into 136 unstranded contexts by taking their median.

#### Ki-67 staining and quantification

Tissue slices were used for Ki-67 staining with respective control tissue (tonsil) appended on the slide. Immunohistochemistry was performed with an automated, validated and accredited staining system (Ventana Benchmark ULTRA, Ventana Medical Systems) using the ultraView universal DAB Detection Kit (Roche, Cat#760-500). In brief, following deparaffinization and heat-induced antigen retrieval the tissue samples were incubated according to their optimized time with the antibody of interest (antibody: MIB1, clone: Ki-67, type: rabbit anti-human, concentration: 0.40 μg/mL, procedure: ultraView CC1 64′, antibody incubation: 32 min, Ventana, Cat#790–4286). Incubation was followed by detection with the secondary antibody included in the ultraview universal kit (multimere), followed by haematoxylin II counter stain for 20 min and then a blue coloring reagent for 8 min according to the manufacturer’s instructions (Ventana Medical Systems Inc., Arizona, USA). The scanned stainings were imported in QuPath v0.5.0 (∗.ndpi files). Samples were imported as Brightfield H-DAB. Regions containing tissue were manually selected and artifacts (gaps and folds) were manually excluded. Within these regions cells were detected using StarDist2D v0.5.0 (dsb2018_heavy_augment; dsb2018_paper; he_heavy_augment; normalizePercentiles: 1, 99; threshold: 0.40; pixelSize: 0.2276; includeProbability: true; cellExpansion: 5). A total of *n* = 20 samples were used for training cell type classification, in which a fraction of cells was selected and annotated as either Ki67+, Ki67-or “other” in case of erythrocytes, air bubbles or other artifacts. A classifier was trained using TrainObjectClassifier on all training cells (Classifier: ANN_MLP; all measurements; all 3 classes). This classifier was exported as *DABnn6* (see KRT). The classifier was applied to all regions: runObjectClassifier("DABnn6");. Data were exported as *measurements*. Snapshots were exported from QuPath to ImageJ v1.54k in which the scale bars were added.

#### Proteomics

From 140 unique resections, two 10μm FFPE tissue sections were used for proteomics analysis. The system used was a Bruker timsTOF attached to an EvoSep system using diaPASEF.^80^^,^^81^ Of these samples, 118 had matching DNA methylation data. Per-peptide intensities were transformed into 8,070 protein-wise intensities using prolfqua.^82^ Raw data was deposited in the PRIDE database under accession PXD070222.^83^ Control entries and entries lacking HUGO gene annotations were excluded. Proteins with more than 60 N/A values were excluded. Intensities were log2 transformed and per-sample robust scaled using median and IQR. In total, 6,540 proteins were available for downstream analysis. Limma^74^ was used for statistical comparison between primary – recurrent, grades and with CGC^ψ^ as a continuous factor. After CGC^ψ^ was scaled using the scale function, protein expression for continuous factors was considered significant with an FDR-adjusted *p* -value<0.01 and |log2FC|>0.5 and for discrete factors with an FDR-adjusted *p* -value<0.01. Pathway enrichment was performed on the up- and down regulated significant differentially expressed proteins using g:Profiler,^84^ using only the proteins with signal as background set. From the manifest file infinium-methylationepic-v-1-0-b5-manifest-file.csv, the column UCSC_RefGene_Name was utilized to map proteomics data to gene-level identifiers. The column UCSC_RefGene_Group served as the source of regulatory element annotations. For downstream analyses, the annotations TSS200 and TSS1500 were merged while annotations corresponding to 3′ UTR and exonBnd were excluded.

### Quantification and statistical analysis

#### R statistical computing

R v4.4.2 was used for data processing, including the limma, uwot, tidyverse, patchwork, survival, survminer, rms, ggrepel, ggbeeswarm, factoextra, pROC, glmnet and ggpubr libraries. The code is freely accessible at the following repository: (https://github.com/yhoogstrate/glass-od). FDR-corrected *p*-values are represented by as asterisks: ⋅ q < 0.05; ∗q < 0.01; ∗∗q < 0.001; ∗∗∗q < 0.0001.

## Supplemental information

Document S1. Figures S1–S9

Table S1. Overview of discovery set

Table S2. Overview of validation set

Table S3. CGCψ covariates, weights, and annotations

Table S4. Ki-67 staining classifier output

Table S5. CDKN2A/B copy-number calls

Table S6. Data overview of oligosarcoma spiked in with non-tumor cortex

Table S7. CpGs tested for treatment effect(s)

Table S8. Overview of TCGA data

Table S9. 1p/19q copy number bins used for tumor purity prediction

Document S2. Article plus supplemental information
