# Supplementary material for: TET CpG sequence-context-specific DNA demethylation shapes progression of IDH-mutant gliomas
Source: Cell Rep Med. 2026 Mar 17;7(3):102682. doi: 10.1016/j.xcrm.2026.102682 (PMC13006442; doi:10.1016/j.xcrm.2026.102682)
Supplement: Document S1. Figures S1–S9 [file mmc1.pdf]

Cell Reports Medicine, Volume 7

## Supplemental information

# TET CpG sequence-

# context-specific DNA demethylation

# shapes progression of IDH-mutant gliomas

**Youri Hoogstrate, Santoesha A. Ghisai, Levi van Hijfte, Rania Head, Iris de Heer, Marta Padovan, Maurice de Wit, Wies R. Vallentgoed, Angelo Dipasquale, Maarten M.J. Wijnenga, Bas Weenink, Rosa Luning, Sybren L.N. Maas, Adela Brzobohata, Michael Weller, Tobias Weiss, Maximilian J. Mair, Anna S. Berghoff, Adelheid Wöhrer, Albert Jeltsch, Johan A.F. Koekkoek, Hans M. Hazelbag, Mathilde C.M. Kouwenhoven, Yongsoo Kim, Bart A. Westerman, Bauke Ylstra, Anneke M. Niers, Kevin C. Johnson, Frederick S. Varn, Roel G.W. Verhaak, Mustafa Khasraw, Martin J. van den Bent, Pieter Wesseling, and Pim J. French**

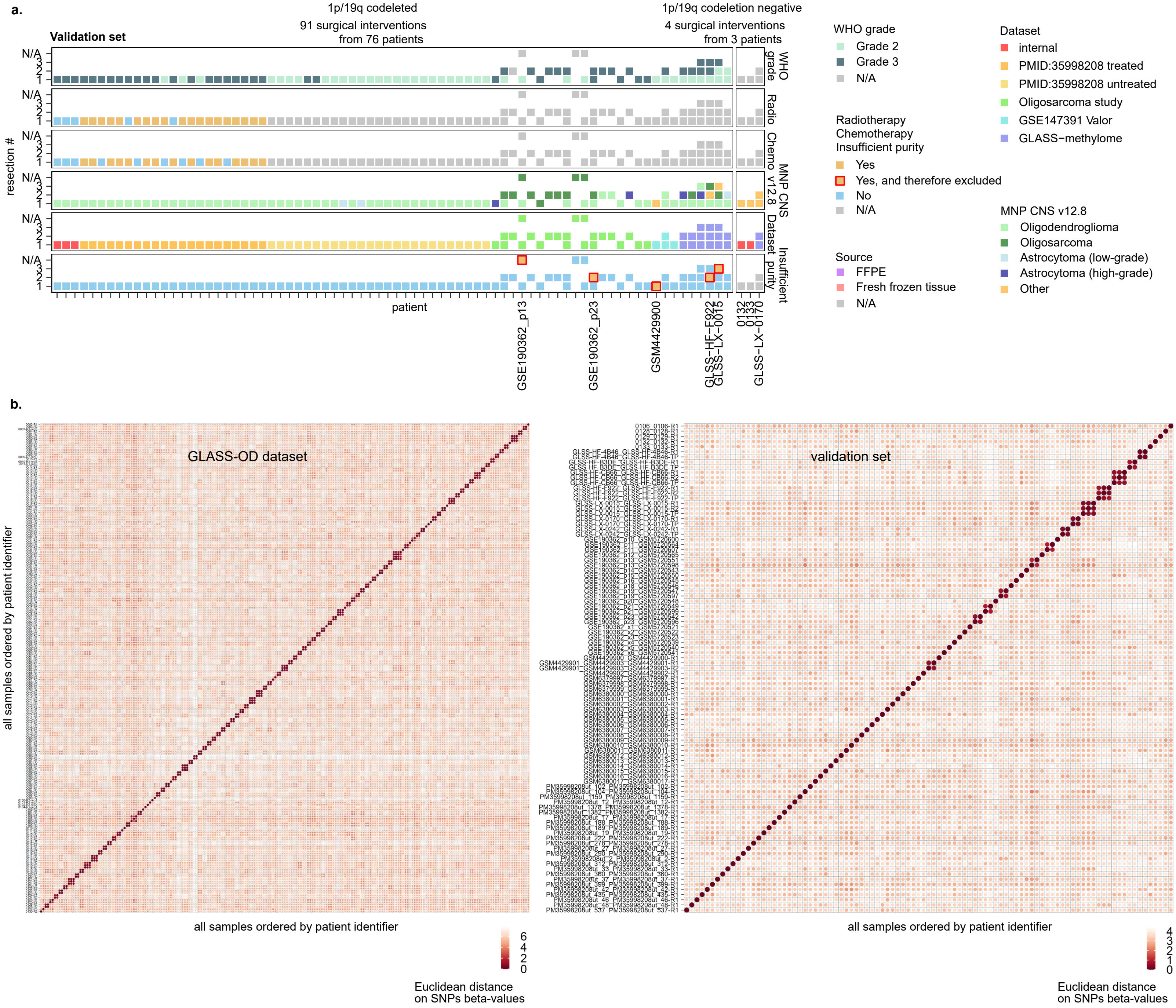
a.
Validation set
1p/19q codeleted
91 surgical interventions
from 76 patients
1p/19q codeletion negative
4 surgical interventions
from 3 patients
N/A
3
2
1
resection #
WHO grade
Radio
Chemo
MNP CNS v12.8
Dataset
Insufficient purity
patient
GSE190362_p13
GSE190362_p23
GSM4429900
GLSS−HF−F922
GLSS−LX−0015
0132
0133
GLSS−LX−0170
WHO grade
Grade 2
Grade 3
N/A
Radiotherapy
Chemotherapy
Insufficient purity
Yes
Yes, and therefore excluded
No
N/A
Source
FFPE
Fresh frozen tissue
N/A
Dataset
internal
PMID:35998208 treated
PMID:35998208 untreated
Oligosarcoma study
GSE147391 Valor
GLASS−methylome
MNP CNS v12.8
Oligodendroglioma
Oligosarcoma
Astrocytoma (low-grade)
Astrocytoma (high-grade)
Other
b.
GLASS-OD dataset
validation set
all samples ordered by patient identifier
all samples ordered by patient identifier
all samples ordered by patient identifier
6
4
2
0
Euclidean distance
on SNPs beta-values
4
3
2
1
0
Euclidean distance
on SNPs beta-values

**Supplementary Fig. 1. Cohort overview and quality control analyses.**

**(a)** Patient overview for the DNA methylation validation dataset. Samples excluded due to low purity are highlighted and samples excluded due to the absence of 1p/19q codeletions are separated.

**(b)** SNP-probe-based fingerprinting. Both axes represent all samples per dataset, ordered by patient identifier and then surgery number. Each dot indicates the Euclidean distance between two samples based on scaled beta-values from SNP probes. Light colors indicate large distances between two samples (low similarity), while dark colors indicate small distances between two samples (high similarity). Samples that belong to the same individual are represented by multiple dark dots.

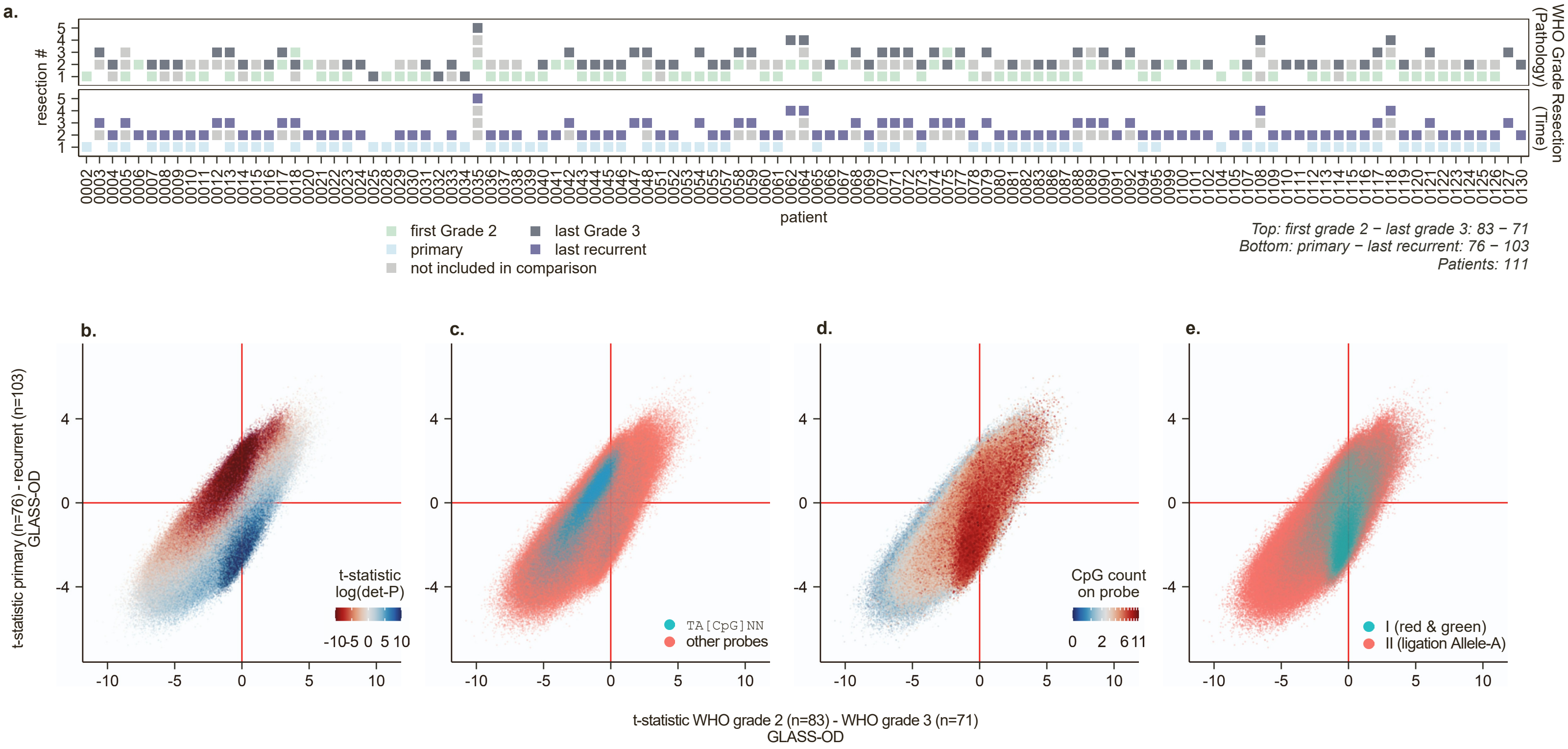
a.
resection #
WHO Grade (Pathology)
Resection (Time)
patient
first Grade 2
primary
not included in comparison
last Grade 3
last recurrent
Top: first grade 2 − last grade 3: 83 − 71
Bottom: primary − last recurrent: 76 − 103
Patients: 111
b.
c.
d.
e.
t-statistic primary (n=76) - recurrent (n=103)
GLASS-OD
t-statistic
log(det-P)
-10-5 0 5 10
TA[CpG]NN
other probes
CpG count
on probe
0 2 6 11
I (red & green)
II (ligation Allele-A)
t-statistic WHO grade 2 (n=83) - WHO grade 3 (n=71)
GLASS-OD

**Supplementary Fig. 2. Notable effect of data quality on DMP outcomes.**

**(a)** Distribution of samples across groups used in the two DMP comparisons. Top: comparison between the first CNS WHO grade 2 samples (light green) and the last WHO grade 3 samples (dark green). Bottom: comparison between primary surgical interventions (light blue) and the last surgical interventions (dark blue).
**(b)** Same integrated DMP plot as in *Fig. 2c*, with probes colored by the t-statistic from an additional DMP model relating CpG methylation levels to the log-transformed percentage of detection p-value failed probes per sample.
**(c)** Same integrated DMP plot as in *Fig. 2c*, with probes colored by having a `TA[CpG]NN` sequence context.
**(d)** Same integrated DMP plot as in *Fig. 2c*, with probes colored by the total number of CpG sites within the probe sequence.
**(e)** Same integrated DMP plot as in *Fig. 2c*, with probes colored by the probe chemistry type.

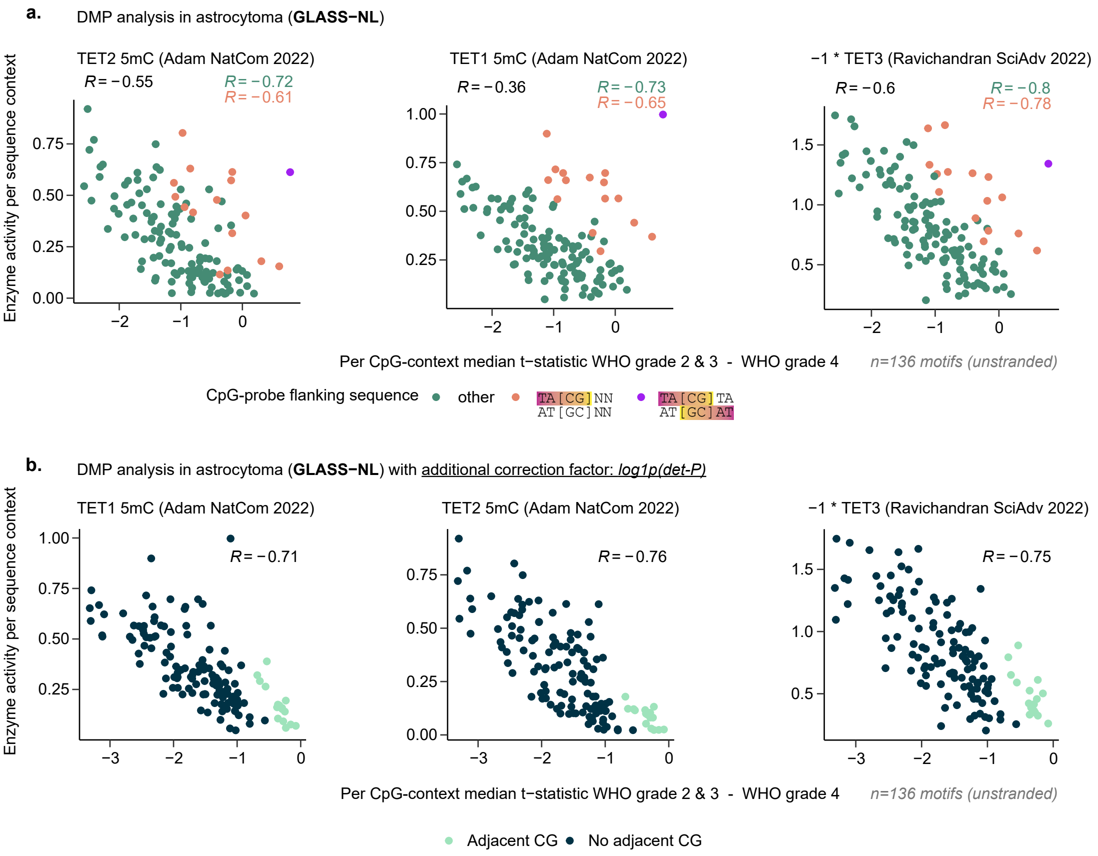
a.
DMP analysis in astrocytoma (GLASS−NL)
TET2 5mC (Adam NatCom 2022)
R = −0.55
R = −0.72
R = −0.61
TET1 5mC (Adam NatCom 2022)
R = −0.36
R = −0.73
R = −0.65
−1 * TET3 (Ravichandran SciAdv 2022)
R = −0.6
R = −0.8
R = −0.78
Enzyme activity per sequence context
Per CpG-context median t-statistic WHO grade 2 & 3 - WHO grade 4
n=136 motifs (unstranded)
CpG-probe flanking sequence
other
TA[CG]NN
AT[GC]NN
TA[CG]TA
AT[GC]AT
b.
DMP analysis in astrocytoma (GLASS−NL) with additional correction factor: log1p(det-P)
TET1 5mC (Adam NatCom 2022)
R = −0.71
TET2 5mC (Adam NatCom 2022)
R = −0.76
−1 * TET3 (Ravichandran SciAdv 2022)
R = −0.75
Enzyme activity per sequence context
Per CpG-context median t-statistic WHO grade 2 & 3 - WHO grade 4
n=136 motifs (unstranded)
Adjacent CG
No adjacent CG

**Supplementary Fig. 3. Changes in methylation are sequence context specific in astrocytoma (GLASS-NL).**

**(a)** Scatterplots showing the median t-statistics per sequence context (x-axis), comparing methylation differences between CNS WHO grade 2 & 3 versus grade 4 astrocytomas, plotted against TET flanking sequence preferences (y-axis). Sequence contexts are color-coded by their sequence matching the quality associated `TA[CpG]` subsequence once, twice (palindromic) or not. Spearman correlation coefficients (ρ) are shown in black for all data points, in orange for contexts with one `TA[CpG]` subsequence, and in green for those not matching.

**(b)** Same as (a) but using a model correcting with *log1p*(fraction det-P failed probes).

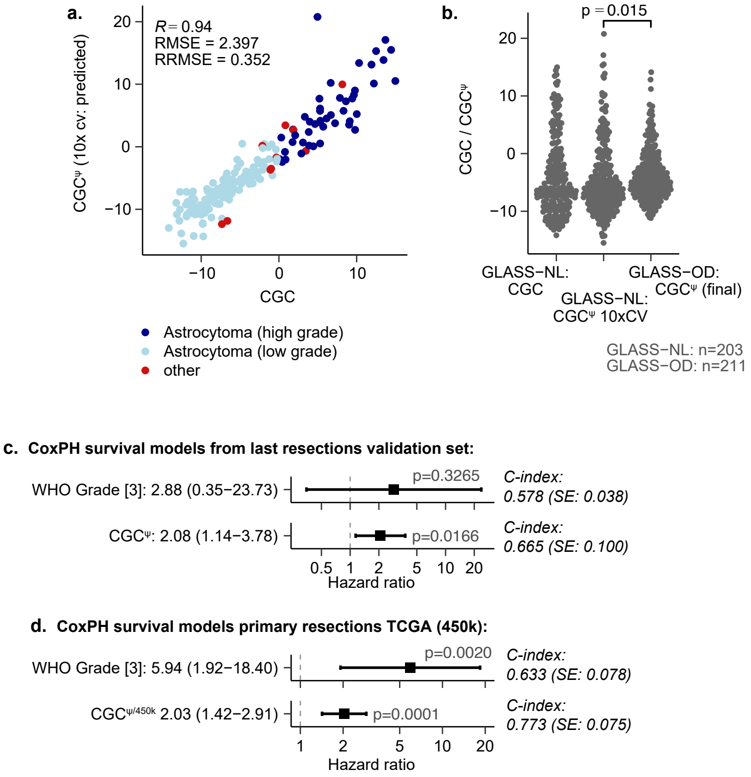
a.
R = 0.94
RMSE = 2.397
RRMSE = 0.352
CGCᴪ (10x cv: predicted)
CGC
−10
0
10
20
Astrocytoma (high grade)
Astrocytoma (low grade)
other
b.
p = 0.015
CGC / CGCᴪ
GLASS−NL: CGC
GLASS−NL: CGCᴪ 10xCV
GLASS−OD: CGCᴪ (final)
GLASS−NL: n=203
GLASS−OD: n=211
c. CoxPH survival models from last resections validation set:
WHO Grade [3]: 2.88 (0.35−23.73)
p=0.3265
C-index: 0.578 (SE: 0.038)
CGCᴪ: 2.08 (1.14−3.78)
p=0.0166
C-index: 0.665 (SE: 0.100)
0.5 1 2 5 10 20
Hazard ratio
d. CoxPH survival models primary resections TCGA (450k):
WHO Grade [3]: 5.94 (1.92−18.40)
p=0.0020
C-index: 0.633 (SE: 0.078)
CGCᴪ/450k 2.03 (1.42−2.91)
p=0.0001
C-index: 0.773 (SE: 0.075)
1 2 5 10 20
Hazard ratio

**Supplementary Fig. 4. CGC$^{\psi}$ model development and multivariate survival analysis.**
**(a)** Scatterplot showing the expected CGC values (actual CGC) versus the observed CGC$^{\psi}$ estimates obtained through 10-fold cross-validation in all GLASS-NL samples.
**(b)** Density scatterplots showing: (left) actual CGC scores in the GLASS-NL dataset, (middle) predicted CGC$^{\psi}$ scores from 10-fold cross-validation in GLASS-NL, and (right) CGC$^{\psi}$ scores from the final model applied to oligodendrogliomas in the GLASS-OD dataset. The difference in CGC$^{\psi}$ between samples from the GLASS-NL and GLASS-OD datasets was test using the Wilcoxon rank-sum test, shown at the top.
**(c)** Forest plots of univariate CoxPH models on overall survival in the last available resections of the *validation set* comparing with CGC$^{\psi}$ (top) and CNS WHO grade (bottom). Hazard ratios are indicated with 95% confidence intervals.
**(d)** Forest plots of univariate CoxPH models on overall survival in the primary resections of oligodendroglioma samples from the *TCGA-LGG dataset* (450k arrays) with CGC$^{\psi/450k}$ (top) and CNS WHO grade (bottom). Hazard ratios are indicated with 95% confidence intervals.

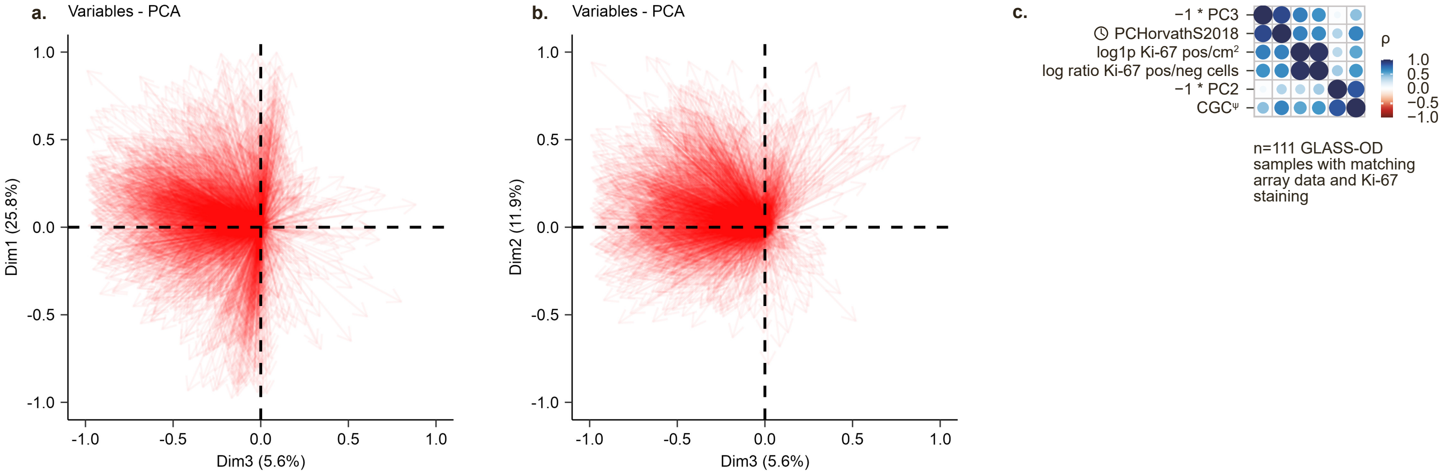
a.
Variables - PCA
Dim1 (25.8%)
Dim3 (5.6%)
1.0
0.5
0.0
-0.5
-1.0
b.
Variables - PCA
Dim2 (11.9%)
Dim3 (5.6%)
c.
−1 * PC3
PCHorvathS2018
log1p Ki-67 pos/cm²
log ratio Ki-67 pos/neg cells
−1 * PC2
CGCᴪ
ρ
1.0
0.5
0.0
−0.5
−1.0
n=111 GLASS-OD samples with matching array data and Ki-67 staining

**Supplementary Fig. 5. CpG probes annotated to belong to polycomb transcription factors contribute to PC3.**
**(a)** Variables factor map for principal components 1 and 3, marking only probes mapped to polycomb transcription factors.
**(b)** Variables factor map for principal components 2 and 3, marking only probes mapped to polycomb transcription factors.
**(c)** Spearman correlation (ρ) based clustering of Ki-67 positive cell ratio and density with other sample parameters, for the n=111 samples with matching Ki-67 staining and DNA methylation arrays.

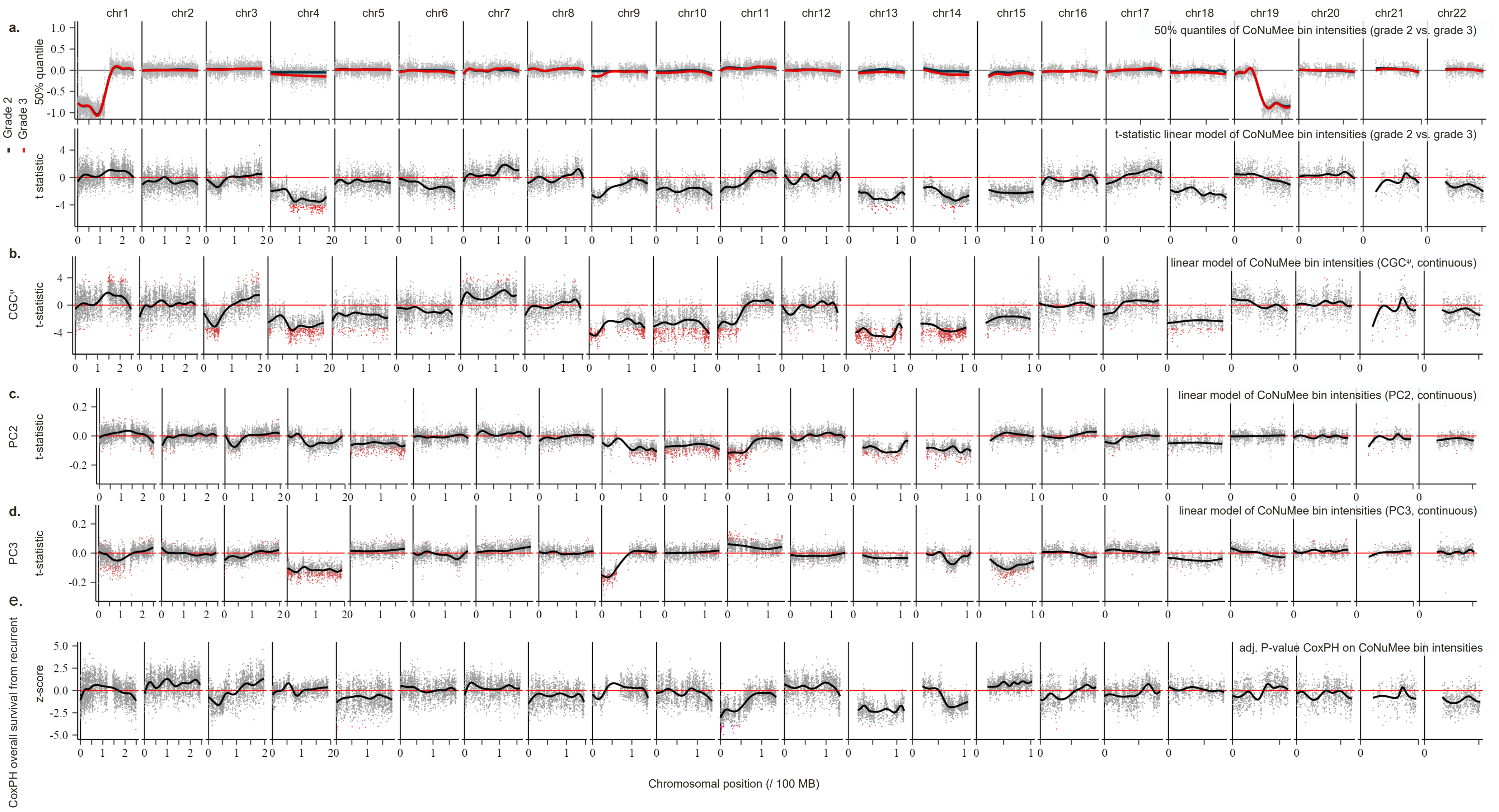

chr1 chr2 chr3 chr4 chr5 chr6 chr7 chr8 chr9 chr10 chr11 chr12 chr13 chr14 chr15 chr16 chr17 chr18 chr19 chr20 chr21 chr22
a.
50% quantiles of CoNuMee bin intensities (grade 2 vs. grade 3)
50% quantile
Grade 2
Grade 3
t-statistic linear model of CoNuMee bin intensities (grade 2 vs. grade 3)
t statistic
b.
linear model of CoNuMee bin intensities (CGCᵠ, continuous)
CGCᵠ
t-statistic
c.
linear model of CoNuMee bin intensities (PC2, continuous)
PC2
t-statistic
d.
linear model of CoNuMee bin intensities (PC3, continuous)
PC3
t-statistic
e.
adj. P-value CoxPH on CoNuMee bin intensities
CoxPH overall survival from recurrent
z-score
Chromosomal position (/ 100 MB)

**Supplementary Fig. 6. Recurrent tumors are characterized by chromosomal losses.**
**(a)** Differences in intensity per CNV bin compared between CNS WHO grades in oligodendroglioma.
**(b)** Differences in CNV bin intensity associated with CGC$^{\Psi}$. T-statistics and q-values (empirical Bayes moderated t-test) are derived from multivariate linear models fitted to CNV bin intensity, including both tumor purity and CGC$^{\Psi}$ (scaled to a standard deviation of 1) as covariates. Regions with a significant association ($q<0.01$) are marked in red.
**(c, d)** Same as (b), but using including tumor purity, PC2 and PC3. Regions with a significant association ($q<0.01$, empirical Bayes moderated t-test) are marked in red.
**(e)** Same as (b), but using a CoxPH model on overall survival at tumor recurrence. Regions with a trend in association ($q>=0.01$ & $q<0.05$, empirical Bayes moderated t-test) are marked in pink.

a.

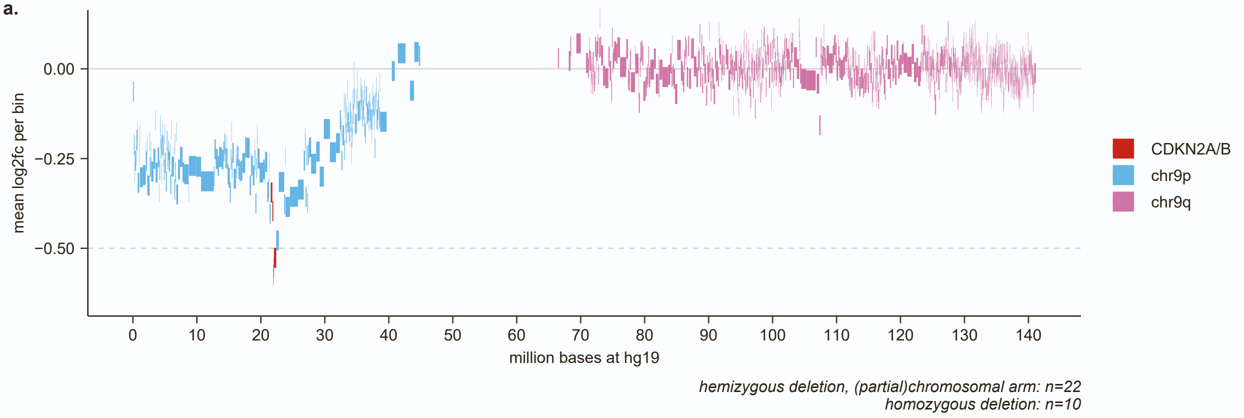
mean log2fc per bin
0.00
−0.25
−0.50
0
10
20
30
40
50
60
70
80
90
100
110
120
130
140
million bases at hg19
hemizygous deletion, (partial)chromosomal arm: n=22
homozygous deletion: n=10
CDKN2A/B
chr9p
chr9q

**Supplementary Fig. 7. The *CDKN2A/B* locus is frequently deleted as a result of partial loss of the chr9p arm.**
**(a)** Detailed copy-number view of chr9, showing the average CoNuMee score of samples with a chr9p arm, partial chr9p arm or a homozygous deletion of *CDKN2A/B* (n=32; **Table S4**).

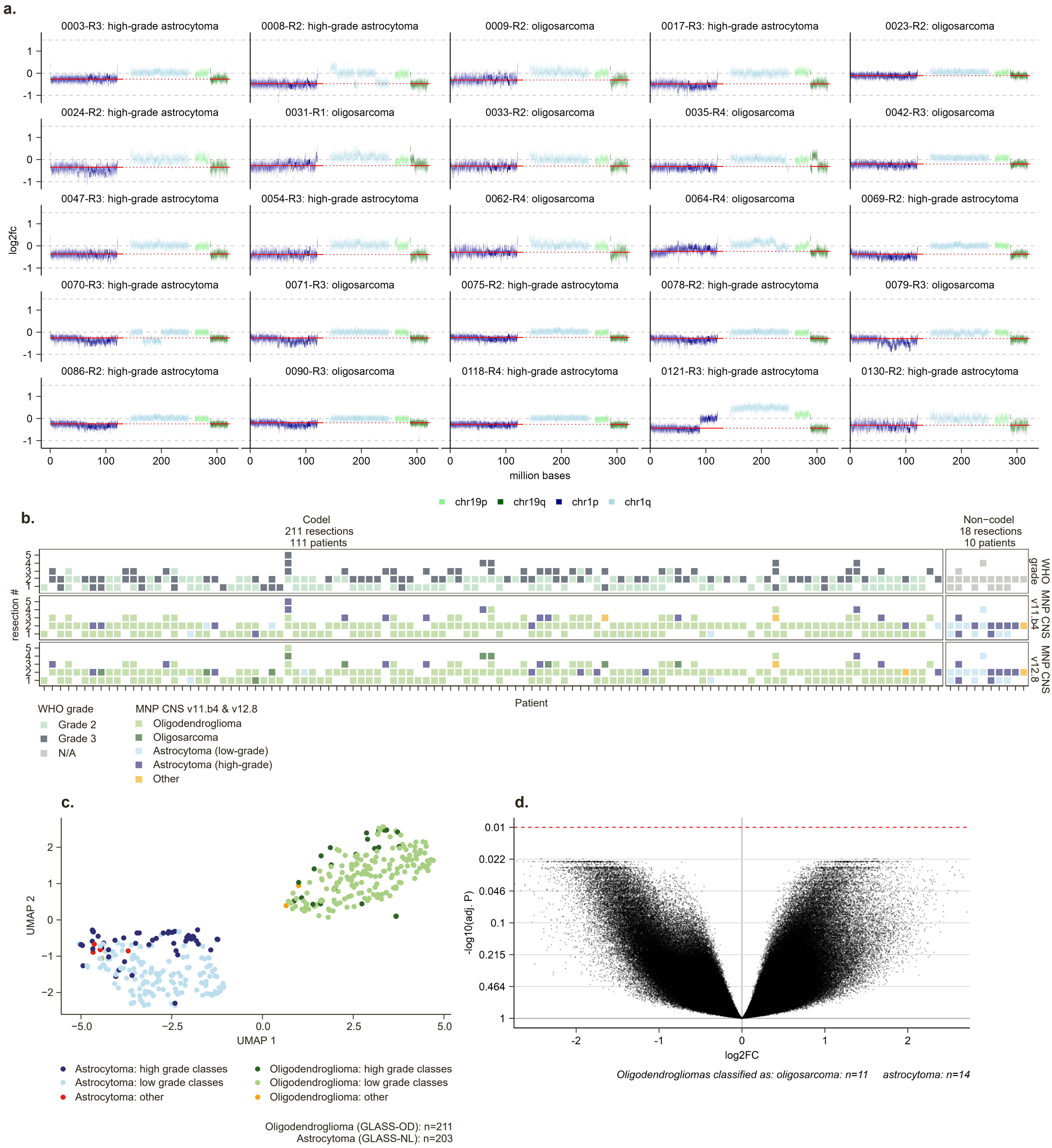

a.
0003-R3: high-grade astrocytoma
0008-R2: high-grade astrocytoma
0009-R2: oligosarcoma
0017-R3: high-grade astrocytoma
0023-R2: oligosarcoma
0024-R2: high-grade astrocytoma
0031-R1: oligosarcoma
0033-R2: oligosarcoma
0035-R4: oligosarcoma
0042-R3: oligosarcoma
0047-R3: high-grade astrocytoma
0054-R3: high-grade astrocytoma
0062-R4: oligosarcoma
0064-R4: oligosarcoma
0069-R2: high-grade astrocytoma
0070-R3: high-grade astrocytoma
0071-R3: oligosarcoma
0075-R2: high-grade astrocytoma
0078-R2: high-grade astrocytoma
0079-R3: oligosarcoma
0086-R2: high-grade astrocytoma
0090-R3: oligosarcoma
0118-R4: high-grade astrocytoma
0121-R3: high-grade astrocytoma
0130-R2: high-grade astrocytoma
log2fc
million bases
chr19p chr19q chr1p chr1q
b.
Codel
211 resections
111 patients
Non−codel
18 resections
10 patients
resection #
WHO grade
MNP CNS v11.b4
MNP CNS v12.8
Patient
WHO grade
Grade 2
Grade 3
N/A
MNP CNS v11.b4 & v12.8
Oligodendroglioma
Oligosarcoma
Astrocytoma (low-grade)
Astrocytoma (high-grade)
Other
c.
UMAP 1
UMAP 2
Astrocytoma: high grade classes
Astrocytoma: low grade classes
Astrocytoma: other
Oligodendroglioma: high grade classes
Oligodendroglioma: low grade classes
Oligodendroglioma: other
Oligodendroglioma (GLASS-OD): n=211
Astrocytoma (GLASS-NL): n=203
d.
-log10(adj. P)
log2FC
Oligodendrogliomas classified as: oligosarcoma: n=11 astrocytoma: n=14

**Supplementary Fig. 8. High grade oligodendrogliomas and astrocytomas remain epigenetically distinct tumor types.**

**(a)** CNV profiles of chromosomes 1 and 19 for GLASS-OD samples classified as either high-grade astrocytoma or oligosarcoma. Chromosome arms are color-coded to highlight the presence of the 1p/19q co-deletion. The x-axis shows genomic distance along chr1 or chr19. The y-axis represents the foldchange as provided by CoNuMee. Red lines indicate tumor purity estimates (based on bin intensities of 1p and 19q).

**(b)** Same patient overview as in *Fig. 1a*, extended with classifications from MNP CNS classifier v11.b4.

**(c)** Uniform Manifold Approximation and Projection (UMAP) of samples from the GLASS-OD (n=211) and GLASS-NL (n=203) datasets, colored by MNP CNS classifier groups: low-grade (light colors; low-grade astrocytoma or oligodendroglioma) and high-grade (dark colors; high-grade astrocytoma or oligosarcoma).

**(d)** Volcano plot showing differential methylation between oligodendrogliomas classified as high-grade astrocytoma (n=14) or oligosarcoma (n=11) by the MNP CNS classifier. Each dot represents a CpG probe. The x-axis represents the $\log_2$ fold change, and the y-axis shows the –log10(FDR-adjusted p-value) (empirical Bayes moderated t-test). The red dashed line represents the adjusted p-value cut-off of 0.01.

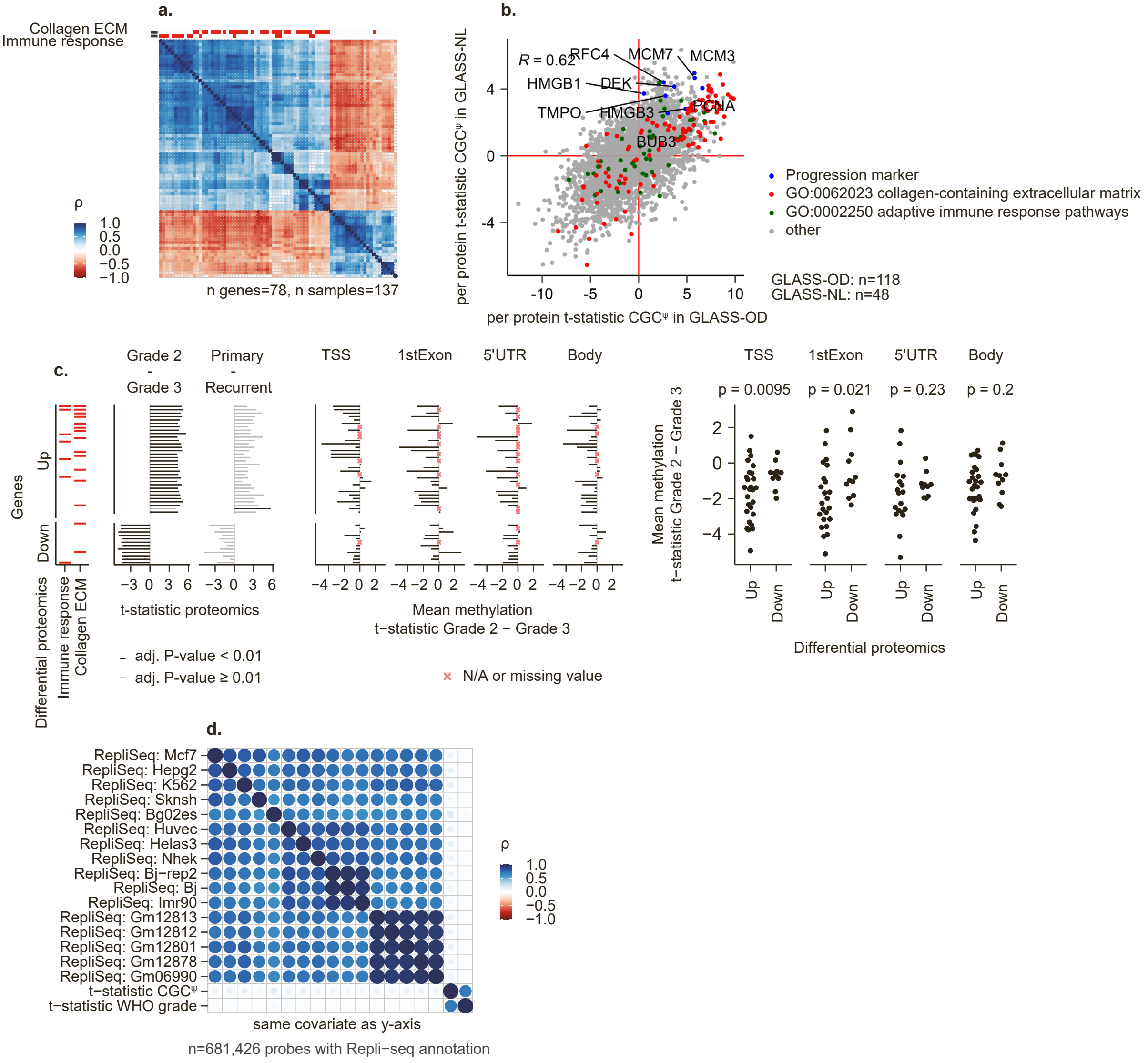

a.
Collagen ECM
Immune response
ρ
1.0
0.5
0.0
−0.5
−1.0
n genes=78, n samples=137
b.
R = 0.62
RFC4
MCM7
MCM3
HMGB1
DEK
TMPO
HMGB3
PCNA
BUB3
per protein t-statistic CGCᴪ in GLASS-NL
per protein t-statistic CGCᴪ in GLASS-OD
Progression marker
GO:0062023 collagen-containing extracellular matrix
GO:0002250 adaptive immune response pathways
other
GLASS-OD: n=118
GLASS-NL: n=48
c.
Grade 2 - Grade 3
Primary - Recurrent
TSS
1stExon
5'UTR
Body
Genes
Up
Down
Differential proteomics
Immune response
Collagen ECM
t-statistic proteomics
Mean methylation t-statistic Grade 2 − Grade 3
adj. P-value < 0.01
adj. P-value ≥ 0.01
N/A or missing value
p = 0.0095
p = 0.021
p = 0.23
p = 0.2
Mean methylation t-statistic Grade 2 − Grade 3
Differential proteomics
d.
RepliSeq: Mcf7
RepliSeq: Hepg2
RepliSeq: K562
RepliSeq: Sknsh
RepliSeq: Bg02es
RepliSeq: Huvec
RepliSeq: Helas3
RepliSeq: Nhek
RepliSeq: Bj−rep2
RepliSeq: Bj
RepliSeq: Imr90
RepliSeq: Gm12813
RepliSeq: Gm12812
RepliSeq: Gm12801
RepliSeq: Gm12878
RepliSeq: Gm06990
t−statistic CGCᴪ
t−statistic WHO grade
ρ
1.0
0.5
0.0
−0.5
−1.0
same covariate as y-axis
n=681,426 probes with Repli−seq annotation

**Supplementary Fig. 9. Collagen ECM and adaptive immune response related protein expression and replication timing in relation to CGC$^{\Psi}$.**

**(a)** Spearman correlation-based clustering of proteins significantly associated with CGC$^{\Psi}$ ($q<0.01$ & $|log2FC|> 0.5$, empirical Bayes moderated t-test). Proteins from the collagen-containing extracellular matrix (GO:0062023) and adaptive immune system reponse (GO:0002250) pathways that according to geneset enrichment analysis were enriched among the upregulated proteins are highlighted in red on top.

**(b)** Intersection of differential protein expression results between oligodendrogliomas from GLASS-OD (fitted to CGC$^{\Psi}$, x-axis) and astrocytomas from GLASS-NL (fitted to CGC, y-axis). Proteins associated with the collagen-containing extracellular matrix (GO:0062023) are marked in red, adaptive immune system reponse (GO:0002250) in green, commonly used proliferation markers are labeled in black and marked in blue. Pearson correlation coefficient (R) is indicated.

**(c)** Integration of proteomics and DNA methylation data in the GLASS-OD dataset. Left panel: (n=44) proteins differentially expressed between CNS WHO grade or primary – recurrent. Genes from the two pathways associated with CGC$^{\Psi}$ are colored in red. For each protein, the t-statistic representing the change in mean methylation between CNS WHO grade 2 and 3 is indicated per annotated regulatory element (TSS, first exon in gene, 5' UTR and gene body). Right panel: The t-statistics comparing the mean methylation for each of the 44 genes between CNS WHO grade 2 and 3, separated by whether they were up- or down regulated in the proteomics data. P-values from t-tests are indicated on top.

**(d)** Correlation between RepliSeq-based replication timing (per genomic bin) and median t-statistics comparing CNS WHO grades in oligodendrogliomas (per same genomic bin). Spearman's rank correlation coefficients (ρ) are indicated.
